# The effect of intolerance of uncertainty on anxiety and depression, and their symptom networks, during the COVID-19 pandemic

**DOI:** 10.1186/s12888-023-04734-8

**Published:** 2023-04-17

**Authors:** Jack L. Andrews, Meiwei Li, Savannah Minihan, Annabel Songco, Elaine Fox, Cecile D. Ladouceur, Louise Mewton, Michelle Moulds, Jennifer H. Pfeifer, Anne-Laura Van Harmelen, Susanne Schweizer

**Affiliations:** 1 University of New South Wales, Sydney, Australia; 2 School of Psychology, University of Adelaide, Adelaide, Australia; 3 University of Pittsburgh, Pittsburgh, USA; 4 University of Oregon, Oregon, USA; 5 Leiden University, Leiden, The Netherlands; 6 University of Cambridge, Cambridge, UK

**Keywords:** Intolerance of Uncertainty, Depression, Anxiety, COVID-19, Network analysis

## Abstract

Individuals vary in their ability to tolerate uncertainty. High intolerance of uncertainty (the tendency to react negatively to uncertain situations) is a known risk factor for mental health problems. In the current study we examined the degree to which intolerance of uncertainty predicted depression and anxiety symptoms and their interrelations across the first year of the COVID-19 pandemic. We examined these associations across three time points (May 2020 – April 2021) in an international sample of adults (*N* = 2087, Mean age = 41.13) from three countries (UK, USA, Australia) with varying degrees of COVID-19 risk. We found that individuals with high and moderate levels of intolerance of uncertainty reported reductions in depression and anxiety symptoms over time. However, symptom levels remained significantly elevated compared to individuals with low intolerance of uncertainty. Individuals with low intolerance of uncertainty had low and stable levels of depression and anxiety across the course of the study. Network analyses further revealed that the relationships between depression and anxiety symptoms became stronger over time among individuals with high intolerance of uncertainty and identified that feeling afraid showed the strongest association with intolerance of uncertainty. Our findings are consistent with previous work identifying intolerance of uncertainty as an important risk factor for mental health problems, especially in times marked by actual health, economic and social uncertainty. The results highlight the need to explore ways to foster resilience among individuals who struggle to tolerate uncertainty, as ongoing and future geopolitical, climate and health threats will likely lead to continued exposure to significant uncertainty.

**Supplementary Information:**

The online version contains supplementary material available at 10.1186/s12888-023-04734-8.

## Introduction

The COVID-19 pandemic has profoundly influenced the health and well-being of individuals across the globe. As with previous pandemics (e.g., SARS, [1]) rising rates of poor mental health, especially, symptoms of depression and generalised anxiety, have been observed globally [2, 3].

One potential factor contributing to the increased rates of depression and generalised anxiety is intolerance of uncertainty. Intolerance of uncertainty refers to an individual’s tendency to react negatively on an emotional, cognitive and behavioural level to uncertain situations and events [4]. Individuals vary in the degree to which they can tolerate uncertainty. Individuals who are the least tolerant (i.e., high in intolerance of uncertainty) are at an increased risk of poor mental health [6, 7], especially generalised anxiety and depression. The COVID-19 pandemic has brought with it significant health, economic and social uncertainties [5]. Individual differences in intolerance of uncertainty may therefore have been particularly predictive of mental health during the pandemic, a time of heightened uncertainty.

The relationship between intolerance of uncertainty and depression and generalised anxiety has been examined during past pandemics. For example, during the swine flu (H1N1 virus) pandemic, intolerance of uncertainty was related to elevated levels of generalised anxiety [8]. This is consistent with cross-sectional evidence demonstrating that during the COVID-19 pandemic individuals with high levels of intolerance of uncertainty were more likely to endorse generalised anxiety and depressive symptoms, relative to individuals with low levels of intolerance of uncertainty [9, 10]. Importantly, symptoms of depression and generalised anxiety often co-occur and comorbidity can lead to more enduring and severe trajectories [6, 7, 11, 12]. Therefore, it is crucial to better understand how factors such as intolerance of uncertainty contribute to risk for depression and generalised anxiety, as well as the associations between their symptoms.

The aim of the present study was two-fold: First, to understand the role intolerance of uncertainty plays in predicting depression and generalised anxiety outcomes across the first year of the COVID-19 pandemic. Second, to examine finer grained associations between intolerance of uncertainty and specific depressive and generalised anxiety symptoms. One promising approach with which to explore these more local associations is through psychometric network analysis, in which the relationships (edges) between individual symptoms (nodes) are modelled [13]. This approach moves beyond traditional categorical approaches to mental health disorders, which assume them to be latent constructs.

In the current study we applied this network approach in two ways. First, we examined the degree to which intolerance of uncertainty moderated the structure and strength of associations between depression and generalised anxiety symptoms, allowing us to see if individuals with high, relative to low, intolerance of uncertainty had more strongly connected symptom networks. In this context, network connectivity refers to the degree to which symptoms (nodes) are correlated with each other in a partial correlation network. Connectivity is an important measure as more highly interconnected symptom networks are vulnerable to a contagion effect in which the severity of one symptom can have a knock-on effect across all other symptoms, potentially leading to higher symptom severities (the “connectivity hypothesis” [14]). Secondly, we incorporated intolerance of uncertainty as a node into a network of depressive and anxiety symptoms. This allowed us to examine how central, or important, intolerance of uncertainty is in connecting symptoms of depression and anxiety, as well as to identify which symptoms it is most strongly associated with.

We tested the three pre-registered hypotheses below (https://osf.io/eyfs2) across three time points from May 2020 to April 2021. To account for variance in health-related uncertainty introduced by the existential threat of the virus we included an index of COVID-19 risk (composite score of morbidity and mortality experienced by the participant and their close kin/friends) in our regression analyses. Additionally, we examined the effect of living in countries with a higher (i.e., USA and UK) compared to those with a lower COVID-19 stringency index (i.e., Australia [15]). The stringency index operationalises governmental measures introduced to curb the spread of COVID-19. These include measures that can be assumed to lead to greater economic uncertainty (e.g., closure of non-essential retail) as well as social uncertainty (e.g., social distancing measures limiting social interactions beyond an individuals’ household).

The study allowed us to test the following hypotheses: First, we predicted that intolerance of uncertainty at time one (T1) would predict depression and generalised anxiety at each assessment time point (*hypothesis 1*). Second, we hypothesized that symptom networks of depression and generalised anxiety would be moderated by intolerance of uncertainty, such that individuals with high (relative to low) intolerance of uncertainty would have more strongly connected symptom networks, and their network connectivity would increase over time (*hypothesis 2*). Third, we hypothesized that intolerance of uncertainty would be a central symptom in a network comprised of intolerance of uncertainty, depression, and generalised anxiety and that its centrality would differ across time (*hypothesis 3*). Finally, we predicted that each of the hypothesized associations (*hypotheses 1–3*) would be stronger in individuals living in countries with a higher COVID-19 stringency index (i.e., UK and US) compared to those living in a country with a lower stringency index (i.e., Australia).

## Methods

### Participants

Participants were recruited online for the COVID-19 Risk Across the Lifespan (CORAL) study from the UK, the US and Australia. The CORAL study is a collaboration across several institutions in these three countries. The CORAL study is an online, longitudinal study which was designed to investigate the effect of the COVID-19 pandemic on individual’s mental health, cognition and social connectedness. Participants were surveyed three times across the course of a year (T1: May fifth, 2020-September thirtieth, 2020; T2: August fifth, 2020-January twenty ninth, 2021; T3: November fifth, 2020-April ninth, 2021). Data were collected using the online Qualtrics survey platform. Recruitment occurred through advertisements on social and conventional media channels. For inclusion in the current study, participants were required to be fluent in English, have no history of traumatic brain injury or neurodevelopmental disorder, be over 18 years of age and have provided data on their level of intolerance of uncertainty. This resulted in a sample of 2087 participants (aged 18–89 years; mean age 41.13; see supplementary materials (SM) for details on the full CORAL sample and participant exclusion details). 91.05% of individuals identified as female (8.2% Male; 0.53% Other; 0.23% Prefer not to say). 86.1% of participants were Caucasian. 45.04% were resident in the UK, 28.75% in the USA and 26.21% in Australia. Access to the CORAL data presented in the current study is by reasonable request from the authors.

### Measures

#### Intolerance of uncertainty

The Intolerance of Uncertainty Scale-Short Form (IUS-12 [16]) was administered to measure responses to uncertainty and ambiguous conditions at T1. The IUS-12 showed good reliability in our sample (Revelle’s *ω*_*t*_ = .94). Scores range from 12 to 60 with higher scores representing higher intolerance of uncertainty.

#### Depression

The Patient Health Questionnaire 8-item Depression Module (PHQ-8^1^ [18]) is an 8-item measure that assesses the severity of depressive symptoms over the previous 2 weeks. Each item is rated from 0 (not at all) to 3 (nearly every day) with higher scores representing higher depressive symptoms. The PHQ-8 showed good reliability in our sample (Revelle’s *ω*_*t*_ = .93 at T1, T2 and T3). The percentage of participants falling within each established cut-off category for depression severity at Time 1 are: None-Minimal (0–4; 30%), Mild (5–9; 29%), Moderate (10–14; 20%), Moderate-Severe (15–19; 14%), Severe (20+; 17%). Time 2: None-Minimal (37%), Mild (32%), Moderate (16%), Moderate-Severe (9%), Severe (6%). Time 3: None-Minimal (36%), Mild (30%), Moderate (18%), Moderate-Severe (10%), Severe (6%).

#### Generalised anxiety

The seven-item Generalized Anxiety Disorder Scale (GAD-7) was used to assess the frequency of anxiety for the past 2 weeks [19, 20]. Each item is scored on a four-point Likert scale from 0 (not at all) to 3 (nearly every day). The GAD-7 showed good reliability in our sample (Revelle’s *ω*_*t*_ at T1 = .96, time two (T2) = .95, time three (T3) = .96). The percentage of participants falling within each established cut-off category for generalized anxiety disorder severity at Time 1 are: None-Minimal (0–4; 37%), Mild (5–9; 28%), Moderate (10–14; 17%), Severe (15+; 18%). Time 2: None-Minimal (46%), Mild (27%), Moderate (15%), Severe (12%). Time 3: None-Minimal (40%), Mild (33%), Moderate (13%), Severe (14%).

#### Country of residence

Participants’ country of residence (Australia, UK, or US) was a proxy for the COVID-19 stringency index. The stringency index is a composite measure of nine indices (i.e., school closures, workplace closures, cancellation of public events, restrictions on gatherings, closure of public transport, stay at home requirements, restrictions on internal movements, international travel controls, and the presence of public information campaigns), computed by the Oxford Coronavirus Government Response Tracker project ([15]; for details see https://ourworldindata.org/covid-government-stringency-index), that indexes the magnitude of a governments' response to COVID-19. From the date the current study commenced until the end of the study period, the average stringency index in the UK was 73.3; in the US, 68.5; and in Australia, 63.6 (on a scale of 0–100, with 100 reflecting the greatest stringency). It should be noted here that the decision to combine the US and UK as high stringency countries was taken at the time of planning these analyses. However, since then the difference in the average stringency index across these countries has increased. We therefore ran additional exploratory analyses including country as a variable with three levels.

### Procedure

The CORAL study was completed online via Qualtrics across three assessment timepoints: T1: May 5, 2020, − September 5, 2020; T2: August 5, 2020, − January 31, 2021, and T3: November 5, 2020 - April 9, 2021. Participants provided informed consent at each assessment. Participants who completed at least 65% of the T1 survey were invited to complete the three- and six-month follow-up assessments. At each time point, attention check items were examined by the researchers. Attention check items are questions with an obvious and correct answer, which are embedded into the survey in order to discern whether or not participants are appropriately reading and responding to the survey questions. Participants who missed or answered more than one attention check item incorrectly were excluded from the study. By participating in the study, every 100th participant was awarded an Amazon gift card with value AUD $100/£50/USD $60 at T1-T3. All participants who completed 100% of the survey at T2 and T3 were additionally awarded an AUD10/£5/USD6 Amazon gift card. All procedures received approval from the University of New South Wales Human Research Ethics Committee (Ref: HC200287).

### Statistical analysis

All analyses were carried out in R (version 4.0.5; R Core Team, 2021). Packages used for subsequent analyses are listed in the SM.

### Hypothesis 1

Linear mixed effects models were used to investigate the relationship between sum scores for intolerance of uncertainty and sum scores for depression (PHQ) and generalised anxiety (GAD) over time (mean and standard deviations at each time point are presented in supplementary table S1). Time, intolerance of uncertainty and country of residence (UK & USA versus Australia) were included as fixed effects and participant ID as a random effect. Missing data was handled using maximum likelihood (ML) estimation which also allows for the comparison of nested models using information criterion (e.g., AIC). Several models were built to predict depression and anxiety scores, separately. The best fitting model was selected based on Akaike Information Criteria (AIC). A lower AIC value indicates a better fitting model. A more complex model was only chosen if it was at least 2 AIC points lower than the simpler model [21]. Sensitivity analyses, controlling for age, gender and COVID risk were also conducted on the best fitting model (Tables S2 and S3). We note that there was no effect of gender in our analyses, and therefore all subsequent analyses were conducted on all participants.

### Hypothesis 2

#### Network estimation, visualisation, and comparison

The combined symptom network of depression and anxiety was estimated with Gaussian Graphical Models (GGM) for each time point and by country [22] (SM include full details on the network estimation procedure). Each network was visualised using the Fruchterman-Reingold algorithm which places the most strongly connected nodes (symptoms) more centrally in the network and the nodes with weaker associations to all other nodes are placed at the periphery [23]. Node predictability was also computed, and visualised on the network, with a more filled ring indicating that more variance (*R*^*2*^) of the node is explained by its correlations with the other nodes in the network that it shares a direct edge with.

The Network Comparison Test (NCT) was used to explore differences between symptom networks. The NCT is a permutation-based hypothesis-test, which assesses differences in the network structure and connectivity of two networks in three ways; first, by testing for network structure invariance; this test compares the difference in the maximal edge weight difference (edge weights refer to the strength of the relationship between two nodes) between the two networks and whether this differs from the largest edge weight difference of two randomly permuted networks (these comparisons will be Bonferroni corrected). Second, and only if the network structure invariance tests reveal a significant difference, individual edge weights are compared between the two networks. Third, global network strength is compared, which is a measure of network connectivity. This test compares the difference in the absolute sum of all the interrelations between the two networks (compared to two randomly permuted networks). Networks were compared using 1000 random permutations.

The NCT was used to compare networks between participants with high and low levels of intolerance of uncertainty across time points. We planned to use standard deviation cut-off criteria to split our sample, however, even with one standard deviation above and below the mean we found extremely uneven group sizes which limits the ability to make accurate network comparisons. Therefore, we conducted a median split on the samples, representing high and low intolerance of uncertainty, with scores falling exactly at the median (35 on our IUS-12 measure and similar to previous studies e.g., [24]) being included in the high group. See Table S14 for sample sizes for each sub-group. The comparisons across levels of intolerance of uncertainty and time were first examined in the full sample and then among adults from countries with a high COVID-19 stringency index (UK, US) and a low COVID-19 stringency index (Australia). Significance was set at *p* < .05.

### Hypothesis 3

#### Network estimation and visualisation

Networks comprising generalised anxiety and depression symptoms, as well as intolerance of uncertainty, were estimated using GGM’s and visualised in the same way as in hypothesis 2.

#### Centrality measures

We computed three centrality measures for each node in the network: strength, betweenness and closeness centrality. Strength centrality refers to the sum of the strength of edges connected to any one node. Betweenness centrality refers to the number of times any one node lies on the shortest path between two other nodes, and closeness centrality is the mean distance of a node from all other nodes in the network.

Prior to interpreting the centrality of each symptom node, we assessed the stability of the strength, betweenness and closeness centrality measures taken from the whole sample, the high stringency index (i.e., US/UK) sample and the low stringency index (i.e., Australia) sample, at T1-T3. Only centrality scores for networks that had a stable correlation stability (CS) coefficient for each measure at 0.5 or above, at each time point, were interpreted [13]. We used a bootstrapped network estimation method, specifying 1000 permutations, to compute the stability measures.

#### Network comparison

For stable centrality measures, NCT was used to assess any changes in the centrality of intolerance of uncertainty between T1, T2 and T3, for the overall sample and for the high and low stringency indices samples. The structure and connectivity of each network were compared, following the same analysis procedure as described for hypothesis 2.

In addition to our pre-registered analyses, we explored which symptoms intolerance of uncertainty were most highly connected in the network at each time point. In doing so, bootstrapped difference tests with 1000 permutations were conducted on the edges connecting intolerance of uncertainty with other symptoms in the network. These analyses were conducted on the full sample at each time point. Significance was set at *p* < .05.

## Results

### Hypothesis 1: predicting depression and generalised anxiety with intolerance of uncertainty

The best fitting model for depression included an interaction between time and intolerance of uncertainty (Table 1). Omnibus tests revealed a significant main effect of intolerance of uncertainty (*F* = (1, 3344.5) = 494.37, *p* < .001). There was no significant main effect of time but there was a significant interaction between time and intolerance of uncertainty (*F* = (1, 1810.5) = 9.78, *p* = .001). The best fitting model for generalised anxiety included an interaction between time and intolerance of uncertainty and country. Omnibus tests revealed a significant main effect of intolerance of uncertainty (*F* = (1, 4406.8) = 446.62, *p* < .001) and time (*F* = (1, 63.7) = 6.74, *p* = .009). There was a significant interaction between time and intolerance of uncertainty (*F* = (1, 132.7) = 14.06, *p* < .001). There were no other significant main or interaction effects. Model estimates are reported in Table 2 and held when controlling for age, gender and COVID-19 risk (Tables S2 and S3).

**Table 1:** Model comparisons for each model predicting depression and generalised anxiety. The best fitting models are highlighted in bold. *Country refers to the comparison between high (UK & USA) and low (Australia) COVID stringency. Bracketed AIC scores for country refer to comparisons made when the UK and USA were not grouped (i.e., UK versus USA versus Australia)

***Model***	***AIC***
Predicting depression	
Time + IU	20,830.41
**Time x IU**	**20,822.65**
Time + IU x Country*	20,828.52 (20,828.22)
Time x IU x Country*	20,822.09 (20,824.80)
Predicting generalised anxiety	
Time + IU	20,437.37
Time x IU	20,418.82
Time + IU x Country*	20,426.72 (20,430.11)
Time x IU x Country*	**20,411.16** (20,417.83)

**Table 2:** Model estimates for the best fitting model predicting depression and generalised anxiety, respectively. In both cases the best fitting model included an interaction between time and intolerance of uncertainty

	***Depression***			***Generalised Anxiety***		
*Predictors*	*Estimates*	*CI*	*p*	*Estimates*	*CI*	*p*
(Intercept)	−2.65	−3.74 – −1.56	**< 0.001**	−4.71	−6.73 – − 2.68	**< 0.001**
Time	0.46	−0.08 – 1.00	0.098	0.63	−0.41 – 1.68	0.235
Intolerance of Uncertainty	0.34	0.31–0.37	**< 0.001**	0.35	0.29–0.41	**< 0.001**
Time x Intolerance of Uncertainty	−0.02	−0.04 – − 0.01	**0.002**	−0.03	− 0.06 – − 0.00	**0.047**
Country	–	–	**–**	− 0.51	−2.87 – 1.85	0.673
Time x Country	–	–	**–**	0.33	−0.88 – 1.54	0.589
Intolerance of Uncertainty x Country	–	–	**–**	0.03	−0.03 – 0.10	0.342
Time x Intolerance of Uncertainty x Country	–	–	**–**	−0.00	−0.04 – 0.03	0.791
Marginal R^2^ / Conditional R^2^	0.251 / 0.735			0.312 / 0.738		

**Fig. 1:**
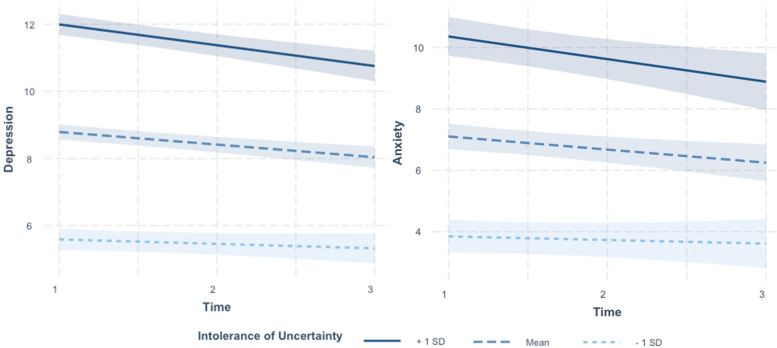
Simple slope analyses resulting from the interaction between time and intolerance of uncertainty, for depression and generalised anxiety. Time 1 occurred between May 5, 2020, and September 30, 2020, time 2 between August 5, 2020, and January 29, 2021, and time 3 between November 5, 2020, and April 9, 2021. When intolerance of uncertainty was high (+ 1SD) individuals’ depressive and anxiety symptoms reduced over time (depression: β = −.62, *p* < .001; anxiety: β = −.65, *p* < .001) but remained higher than individuals with average and low intolerance of uncertainty. Individuals with average intolerance of uncertainty also reported declining depressive and generalised anxiety symptoms over time (depression: β = −.38, *p* < .001; anxiety: β = −.30, *p* < .001), but they remained higher than those of individuals with low intolerance of uncertainty. Individuals with low intolerance of uncertainty reported stable and low levels of depressive and generalised anxiety symptoms over time (depression: β = −.13, *p* = .22; anxiety: β = .04, *p* = .69) (Fig. 1)

The interaction between time and intolerance of uncertainty for models predicting both depression and generalised anxiety were deconstructed with simple slopes analyses (Fig. 1). These showed significant reductions in generalised anxiety and depressive symptoms over time in individuals with high and average levels of intolerance of uncertainty. Individuals low in intolerance of uncertainty reported low levels of symptoms across time.

### Hypothesis 2: the moderating role of intolerance of uncertainty on depression and generalised anxiety symptom networks

We first explored network differences between individuals with high versus low intolerance of uncertainty at each time point. We observed no significant differences in the network structure, or global strength of the networks split, at T1 or T3. There was, however a significant difference in global strength at T2, such that individuals with high intolerance of uncertainty had more strongly connected symptom networks (difference .52, *p* = .02; Table S3).

We then compared the networks separately for individuals high and low in intolerance of uncertainty across time and also by country. For individuals high in intolerance of uncertainty there was a significant difference in global strength, between T1 and T2, such that the symptom network of depression and anxiety was more strongly connected at T2 versus T1 (difference .50, *p* = .01; Table S4). There were no additional differences across timepoints, or by country for individuals high in intolerance of uncertainty. Fig. S1 represents the network diagram and Table S5 and Fig. S2 report node predictability and centrality values for each symptom. In individuals low in intolerance of uncertainty there were no significant differences (Table S4).

### Hypothesis 3: the centrality of intolerance of uncertainty in a network model of depression and generalised anxiety

#### Differences in network structure and connectivity

We next included intolerance of uncertainty as a node within the network model of depression and generalised anxiety. Among individuals from a high stringency index country (UK & US) there was a significant difference in the network structure between T1 and T3 (maximum edge weight difference .17, *p* = .04; Table S6). Posthoc edge weight difference tests found that this was driven by change across four edges (between loss of interest (“PHQ 1”) and Psychomotor problems (“PHQ 8”); Appetite problems (“PHQ 5”) and Feeling nervous (“GAD 1”); Loss of interest (“PHQ 1”) and Worrying too much (“GAD 3”); Worrying too much (“GAD 3”) and Feeling afraid (“GAD 7”), however, these differences did not survive correction for multiple comparisons (Table S7). All other comparisons were non-significant (Table S6).

##### Differences in centrality of intolerance of uncertainty across time

The CS-coefficients for betweenness centrality and closeness centrality were largely unstable (CS-coefficient < .05). Therefore, we were unable to make comparisons across time for these measures. Strength centrality was stable across all three time points for the full sample (CS-coefficient > .05), but unstable for all sub-samples at T2 (Table S8 and Fig. S3). Therefore, Comparisons of strength centrality across time are included for the full sample only.

**Fig. 2:**
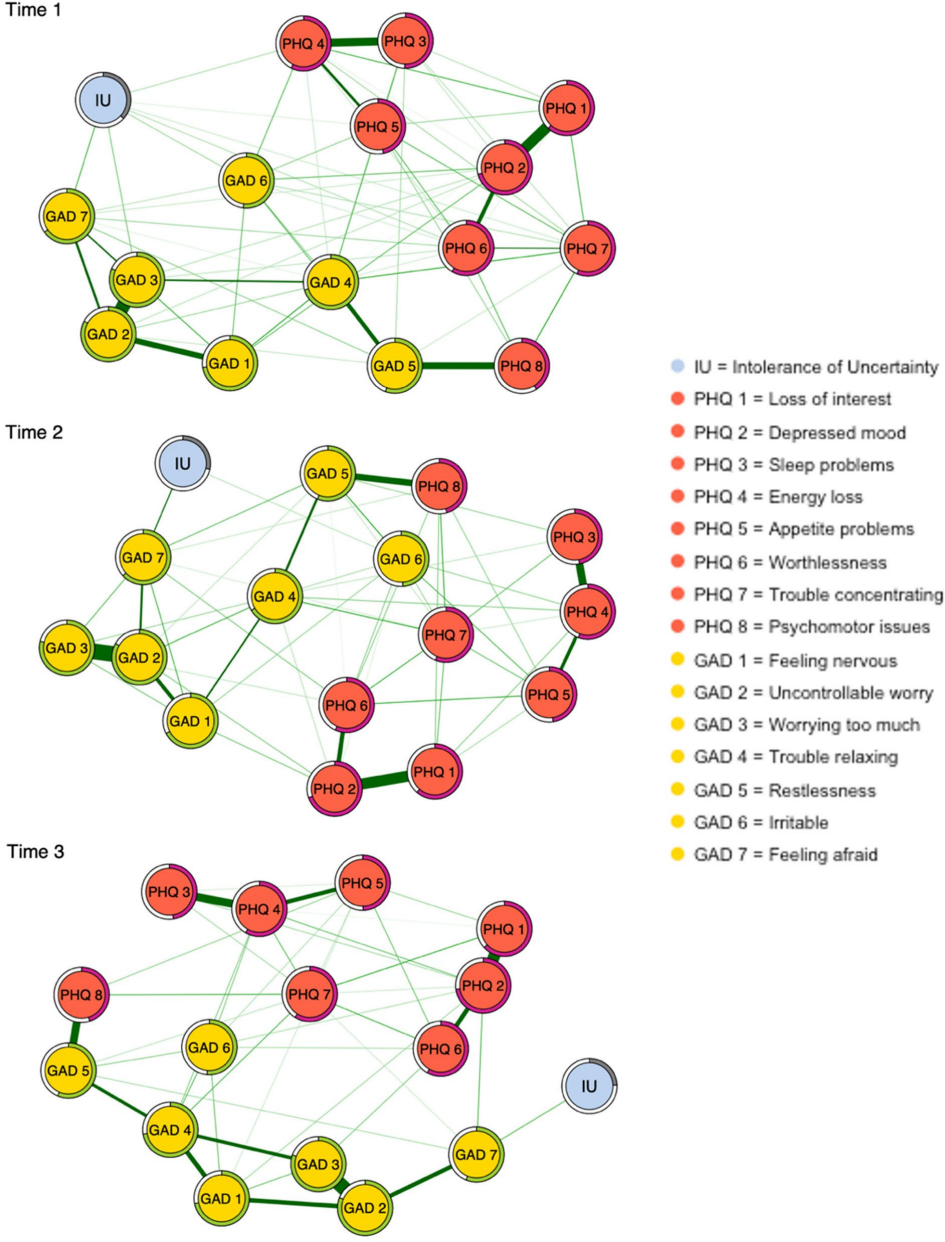
Network diagram of a model comprised of intolerance of uncertainty (sum score of IUS-12) and depression (PHQ-8) and generalised anxiety (GAD-7) symptoms at T1, T2 and T3 for the full sample. The thickness and saturation of the edge colour suggesting the magnitude of the association with a thicker and more saturated edge indicating a stronger correlation between two nodes. Green edges indicate positive associations and red negative. The ring-shaped pie charts around the nodes visualise the node predictability, with a more filled ring indicating that more variance of the node is predicted by its correlations with the other nodes in the network that it shares a direct edge with (Table S10 for all values). Time 1 occurred between May 5, 2020, and September 5, 2020, time 2 between August 5, 2020, and January 31, 2021, and time 3 between November 5, 2020, and March 30, 2021

**Fig. 3:**
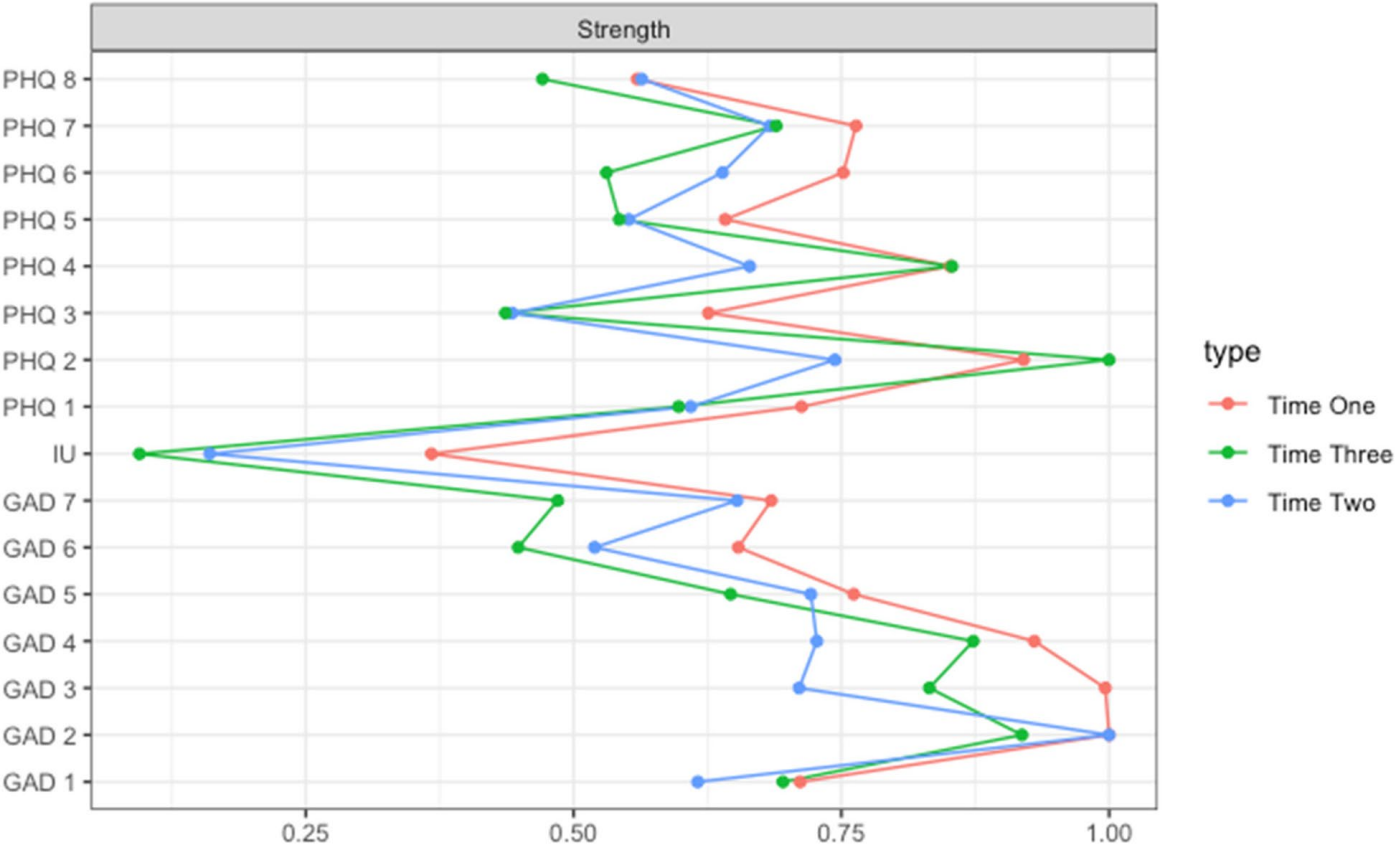
Strength centrality of all nodes at T1, T2 and T3 for the full sample. Strength centrality values are depicted on the x axis and are shown on a relative scale, from 0 (the lowest) to 1 (the highest). Time 1 occurred between May 5, 2020, and September 5, 2020, time 2 between August 5, 2020, and January 31, 2021, and time 3 between November 5, 2020, and March 30, 2021

As preregistered, we were primarily interested in examining differences in the centrality of intolerance of uncertainty across time and these are reported below. Additionally, differences in the strength centrality of each depression and anxiety symptom are reported in Table S6. The strength centrality of intolerance of uncertainty was significantly different between T1 and T3 (difference = .12, *p* = .02), such that intolerance of uncertainty became less central over time (Figs. 2 and 3; Table S9). There was no significant difference in the strength centrality of intolerance of uncertainty between T1 and T2 (difference = .09, *p* = .08), or T2 and T3 (difference = .04, *p* = .58 (Figs. 2 and 3; Table S9). We further computed the node predictability of intolerance of uncertainty at each time point. Intolerance of uncertainty had the highest predictability at T1 (*R*^*2*^ = .37), and this reduced at T2 (*R*^*2*^ = .29) and T3 (*R*^*2*^ = .24) (see Table S10 for the predictability of each node in the network across timepoints).

##### Within network edge weight differences

Intolerance of uncertainty showed the largest unique relationship with item “GAD 7”: ‘feeling afraid as if something awful might happen’ (T1: edge weight = .12, 95% CI = [.07, .17]; T2: edge weight = .14, 95% CI = [.07, .25]; T3: edge weight = .10, 95% CI = [.01, .22]). At T1 the edge between intolerance of uncertainty and “GAD 7” was significantly larger than the edges between intolerance of uncertainty and all other variables (*p* < .05) except for “GAD 3” ‘worrying too much’ and “GAD 6” ‘irritability’ (*p* > .05; Table S11). Whilst the strongest edge with intolerance of uncertainty at T2 and T3 was also with “GAD 7”, this edge was not significantly stronger than the edge between intolerance of uncertainty and all other symptoms in the network (*p* > .05; Tables S12 and S13).

To investigate whether the strength of the association between intolerance of uncertainty and GAD 7 decreased from T1-T3 due to the items’ concurrent measurement at T1, we explored the moderating effect of time on the relationship between intolerance of uncertainty and feeling afraid within T1. We found a significant interaction between intolerance of uncertainty and time (*F* = (1, 5.77) = 7.36, *p* = .007), such that the relationship between intolerance of uncertainty and feeling afraid was weaker among individuals who completed the survey later, indicating that this relationship was stronger at the start of the pandemic and became weaker over time within T1 (see Table S15 and Fig. S4).

## Discussion

We examined the relationship between individuals’ ability to tolerate uncertainty and symptoms of depression and generalised anxiety across the first year of the COVID-19 pandemic. Adults who reported high levels of intolerance of uncertainty had the highest rates of depression and generalised anxiety symptoms at each time point, relative to individuals who reported being better able to tolerate uncertainty. Individuals high in intolerance of uncertainty showed a reduction in symptoms of depression and generalised anxiety across the first year of the pandemic. In contrast, adults with low levels of intolerance of uncertainty reported lower rates of depression and generalised anxiety symptoms, which remained low and stable across time. These findings are consistent with previous cross-sectional findings during the COVID-19 pandemic suggesting that high intolerance of uncertainty is associated with poor mental health outcomes (i.e., elevated symptom scores on established measures of depression and generalised anxiety) [9, 10].

Our finding that adults who indicated that they were able to tolerate uncertainty reported consistently low levels of depression and generalised anxiety across the course of the first year of the COVID-19 pandemic leads us to ask: how malleable is our ability to tolerate uncertainty? Despite intolerance of uncertainty typically being viewed as a trait measure [6], increasing people’s capacity to tolerate uncertainty may be a promising way to help foster metal health resilience, particularly during times of significant ambiguity such as the COVID-19 pandemic. Indeed, a few studies have indicated that intolerance of uncertainty is malleable, and reducing intolerance of uncertainty is related to positive mental health outcomes (i.e., improvements in symptoms of depression and generalised anxiety) [7, 25]. For example, in one study intolerance of uncertainty decreased following a 12-week group CBT intervention for generalised anxiety. In turn, the degree to which individuals’ levels of intolerance of uncertainty decreased was predictive of improvements in generalised anxiety symptoms [26]. Therefore, interventions that have the ability to foster tolerance of uncertainty may hold promise in improving mental health symptoms.

We further found that at our second data assessment time point, intolerance of uncertainty moderated the association between depression and generalised anxiety symptoms such that individuals with high, relative to low, levels of intolerance of uncertainty reported greater symptom connectivity (increased overall strength of symptom associations). Moreover, among adults with high intolerance of uncertainty, symptom connectivity became stronger from T1 to T2. This result is interesting given that overall rates of depression and generalised anxiety reduced over time in these adults. Increased connectivity between symptoms over time, despite declining overall severity of depression and generalised anxiety symptoms [27, 28], has been proposed to reflect a ‘positive spirals’ effect. Positive spirals refer to the effect whereby improvements in one symptom lead to improvements in another. Therefore, whilst overall symptom levels decline, the associations between each symptom may increase [28]. Counter to our hypothesis, we found no effect of country, only observing this effect across the full sample. This may in part be due to lack of power to detect effects given the significantly reduced sample sizes as a result of grouping individuals by country of residence and time point. However, levels of self-reported uncertainty tolerance did not differ across the three countries despite varying levels of governmental stringency indices. Another factor that may also account for these findings, is that we administered a trait, not state, measure of intolerance of uncertainty.

When intolerance of uncertainty was included in our network model of depression and anxiety, we found that it became less central in the network over the course of the pandemic. This finding was further mirrored by the declining degree of variance in intolerance of uncertainty that was explained over time by the symptoms in the network it shared direct associations with. Nonetheless, intolerance of uncertainty assessed six-months prior still accounted for nearly a quarter (24%) of the variance in symptoms of anxiety and depression at the third assessment time point. When examining associations with specific symptoms, intolerance of uncertainty was most strongly associated with feeling afraid. Indeed, intolerance of uncertainty is theorised to represent an underlying fear of the unknown [29]. In the current context, individuals with high levels of intolerance of uncertainty may have been more likely than others to have a high degree of fear about the potential outcomes of the COVID-19 pandemic. One possible interpretation of our finding that the association between feeling afraid and intolerance of uncertainty decreased over time, is that the first months of the pandemic represented the greatest degree of fear of the unknown, as the causes and consequences of the outbreak were still very uncertain. Moreover, exploring this association in T1 only, revealed that the relationship between these variables decreased over time. This gives us confidence that the finding is not merely accounted for by the concurrent measurement of intolerance of uncertainty and mental health at T1.

Further, we found that two items measuring worry held the highest strength centrality at time point one and two. These items were closely connected to feeling afraid, and this finding points to worry as a key symptom of generalised anxiety during the initial months of the pandemic. By the third time point, the centrality of worry was lower than it was at time one and two. However, depressed mood become the more central symptom in the network at time three, indicating its salience in maintaining other depressive and generalised anxiety symptoms as the pandemic progressed. Whilst centrality offers a relative measure of how connected a symptom is, predictability offers an absolute measure and is computed as the shared variance of each symptom node with all its adjacent symptoms. Across each time point, both items tapping into worry held the highest predictability scores. Given that predictability provides an ability to quantify the influence that any single symptom may have on its neighbours, this points to potential targets for intervention [30]. Therefore, both personalised and public health interventions during periods of high uncertainty, as is characteristic of a pandemic, might seek to focus on targeting worry.

Our study should be considered within the context of a number of limitations. Our sample was predominantly comprised of adults who self-identify as female, and despite finding no effect of gender, caution should be taken when generalising beyond the present sample. For example, it is possible that the effects demonstrated here may not generalise to all population sub-groups, necessitating further work to explore how intolerance of uncertainty may influence mental health outcome among various at-risk groups. Additionally, whilst we controlled for COVID risk (i.e., exposure to morbidity and mortality among friends/family) our measure did not include other important factors such as being a first-line healthcare professional or having medical comorbidities that increased risk from COVID-19. We had also planned to interpret changes in the betweenness centrality of intolerance of uncertainty across time. This would have allowed us to make inferences regarding the role it plays in connecting otherwise disparate symptoms (i.e., providing us with more detailed information on its role in the co-morbid nature of depression and generalised anxiety symptoms). However, this measure was unstable preventing us from conducting these analyses. Betweenness centrality is highly sensitive to sample size [31] and became less stable over time as our sample sizes decreased. To ameliorate these issues, future studies should look at ways to reduce rates of attrition, or perhaps pool data across multiple studies in order to be able to make these inferences.

Lastly, given the correlational nature of our study we are unable to infer causality between the constructs we have studied. However, further work should seek to model possible causal pathways between intolerance of uncertainty and depression and generalised anxiety symptoms (e.g., through the use of Directed Acyclic Graphs). For example, this approach has been used to show the possible causal pathway between affective depressive symptoms (feelings of worthlessness, depressed mood and loss of interest) and risk of suicide [32]. In our study we also found that these three symptoms of depression clustered at each time point, and whilst we were unable to explore the association with suicide risk in the current study, future work should prioritise unpacking causal links between intolerance of uncertainty, depressive and generalised anxiety symptoms and suicide risk.

## Conclusion

Our study showed that across the first year of the COVID-19 pandemic, individuals’ perception of their ability to tolerate uncertainty predicted depression and generalised anxiety outcomes and moderated the relationships between depression and generalised anxiety symptoms. Further, intolerance of uncertainty showed the largest association with feeling afraid, relative to all other generalised anxiety and depressive symptoms, which was especially strong towards the start of the pandemic. Our findings sit within a broader literature identifying intolerance of uncertainty as a risk factor for mental health difficulties. One important implication of our findings is that interventions aimed at fostering tolerance towards uncertainty may hold promise in reducing the mental health burden associated with the inevitable occurrence of future global pandemics.

## Supplementary Information

**Additional file 1: Table S1.** Mean and standard deviations of depression (PHQ-8), Anxiety (GAD-7) and Intolerance of Uncertainty (IUS-12) at each time point. **Table S2.** Estimates for models predicting depression when controlling for age, gender and covid risk, respectively. **Table S3.** Estimates for models predicting anxiety when controlling for age, gender and covid risk, respectively. **Table S4.** Results from the Network Comparison Test, assessing differences in network structure invariance and global strength invariance. **Fig. S1.** Network diagram of depressive and anxiety symptoms for the full sample at T1. **Table S5.** Node predictability at T1 for the full sample. **Fig. S2.** The relative scores for strength and closeness centrality are plotted for each node within the network at T1 for the full sample. **Table S6.** Results from the Network Comparison Test, assessing differences in network structure invariance and global strength invariance, across time and by country. **Table S7.** Post-hoc edge weight differences conducted on the edges of a network model comprised of intolerance of uncertainty (sum score of IUS-12) and depression (PHQ-8) and anxiety (GAD-7) symptoms, between T1 and T3 from adults in the UK and USA. **Table S8.** Stability Co-efficients for betweenness, closeness and strength centrality, computed on a network model comprised of intolerance of uncertainty (sum score of IUS-12) and depression (PHQ-8) and anxiety (GAD-7) symptoms, across time and by country. **Fig. S3.** Stability plots for betweenness, closeness and strength centrality by sample and time point. **Table S9.** Differences in strength centrality of each node, computed on a network model comprised of intolerance of uncertainty (sum score of IUS-12) and depression (PHQ-8) and anxiety (GAD-7) symptoms, from the full sample across time. **Table S10.** Node predictability of each symptom, computed on a network model comprised of intolerance of uncertainty (sum score of IUS-12) and depression (PHQ-8) and anxiety (GAD-7) symptoms, from the full sample across time. **Table S11.** Edge weight differences between intolerance of uncertainty and each other symptom node in the network, computed on a network model comprised of intolerance of uncertainty (sum score of IUS-12) and depression (PHQ-8) and anxiety (GAD-7) symptoms, from the full sample at T1 (between May 5, 2020, and September 30, 2020). **Table S12.** Edge weight differences between intolerance of uncertainty and each other symptom node in the network, computed on a network model comprised of intolerance of uncertainty (sum score of IUS-12) and depression (PHQ-8) and anxiety (GAD-7) symptoms, from the full sample at T2 (between August 5, 2020, and January 29, 2021). **Table S13.** Edge weight differences between intolerance of uncertainty and each other symptom node in the network, computed on a network model comprised of intolerance of uncertainty (sum score of IUS-12) and depression (PHQ-8) and anxiety (GAD-7) symptoms, from the full sample at T3 (between November 5, 2020, and April 9, 2021). **Table S15.** Model estimates from a model predicting felling afraid (GAD 7) with an interaction between intolerance of uncertainty and survey completion date (time). **Fig. S4.** The moderating effect of survey completion date (time) on the relationship between intolerance of uncertainty and feeling afraid (GAD 7).

## Data Availability

Data is available on reasonable request from the corresponding author.

## Footnotes

1 The PHQ-8 is derived from the nine-item PHQ-9, excluding item nine which assesses suicidality [17]. The item was excluded as risk could not be managed in this online survey across three different countries.
