# Supplementary material for: The effect of intolerance of uncertainty on anxiety and depression, and their symptom networks, during the COVID-19 pandemic
Source: BMC Psychiatry. 2023 Apr 17;23:261. doi: 10.1186/s12888-023-04734-8 (PMC10109227; doi:10.1186/s12888-023-04734-8)
Supplement: Supplementary file 1 — Additional file 1: Table S1. Mean and standard deviations of depression (PHQ-8), Anxiety (GAD-7) and Intolerance of Uncertainty (IUS-12) at each time point. Table S2. Estimates for models predicting depression when controlling for age, gender and covid risk, respectively. Table S3. Estimates for models predicting anxiety when controlling for age, gender and covid risk, respectively. Table S4. Results from the Network Comparison Test, assessing differences in network structure invariance and global strength invariance. Fig. S1. Network diagram of depressive and anxiety symptoms for the full sample at T1. Table S5. Node predictability at T1 for the full sample. Fig. S2. The relative scores for strength and closeness centrality are plotted for each node within the network at T1 for the full sample. Table S6. Results from the Network Comparison Test, assessing differences in network structure invariance and global strength invariance, across time and by country. Table S7. Post-hoc edge weight differences conducted on the edges of a network model comprised of intolerance of uncertainty (sum score of IUS-12) and depression (PHQ-8) and anxiety (GAD-7) symptoms, between T1 and T3 from adults in the UK and USA. Table S8. Stability Co-efficients for betweenness, closeness and strength centrality, computed on a network model comprised of intolerance of uncertainty (sum score of IUS-12) and depression (PHQ-8) and anxiety (GAD-7) symptoms, across time and by country. Fig. S3. Stability plots for betweenness, closeness and strength centrality by sample and time point. Table S9. Differences in strength centrality of each node, computed on a network model comprised of intolerance of uncertainty (sum score of IUS-12) and depression (PHQ-8) and anxiety (GAD-7) symptoms, from the full sample across time. Table S10. Node predictability of each symptom, computed on a network model comprised of intolerance of uncertainty (sum score of IUS-12) and depression (PHQ-8) and anxiety (GAD-7) sy [file 12888_2023_4734_MOESM1_ESM.docx]

**Supplementary material**

**Participant exclusion details**

The CORAL study recruited 10111 individuals over the age of 11-years-old. To be included in the current study participants needed to be fluent English-speakers (defined as native or having lived and worked in an English-speaking country for 5 years or more), be 11 years old or more, resident in the UK, the US or Australia, have no history of traumatic brain injury or neurodevelopmental disorder (as the wider study also included a cognitive task) and provide informed consent. Individuals under the age of 18 years additionally needed to provide parental consent. 8024 participants had to be excluded: One participant withdrew from the survey; 9 duplicate participants were excluded; 3353 participants were excluded due to not providing consent to participate; 343 participants were excluded due to being under 18-years-old; 737 participants were excluded due to not answering the intolerance of uncertainty scale item; 39 participants were excluded due to not answering the age item; 257 participants were excluded due to parents not consenting for their child; 3092 participants were excluded who were under 18-years-old and accessed the survey without an activation code; two participants were excluded due to lack of English fluency; seven participants were excluded due to reporting a history of neurodevelopmental/neurological disorder; 28 participants were excluded due to reporting a history of TBI; 77 participants were excluded due to reporting a history of neurological disorder; seven participants were excluded due to not living in the UK, US or Australia; three participants were excluded due to lack of capacity to consent; 59 participants were excluded due to not responding to all eligibility items; three participants were excluded due to under the age of 11; eight participants were excluded due to answering more than one attention item incorrectly.

**Primary R packages used**

Lme4 (Version 1.1-26) was used to run the linear mixed models.

Interactions (Version 1.1.3) was used to visualise interactions form each mixed model.

Mgm (Version 1.2-12) was used to estimate the network models.

Igraph (version 1.2.6) was used to visualise the network models.

NetworkComparisonTest (Version 2.2.1) was used to compare network models.

Bootnet (Version 1.4.3) was used to bootstrap network estimations so that centrality measures could be assessed.

|  | *Mean (SD)* | | |
| --- | --- | --- | --- |
|  | *Time 1* | *Time 2* | *Time 3* |
| Depression | 8.92 (6.20) | 7.66 (6.00) | 7.97 (6.06) |
| Anxiety | 7.84 (6.12) | 6.42 (5.68) | 7.00 (5.89) |
| Intolerance of Uncertainty | 35.14 (10.30) | NA | NA |
| ***Table S1.*** *Mean and standard deviations of depression (PHQ-8), Anxiety (GAD-7) and Intolerance of Uncertainty (IUS-12) at each time point.* | | | |

**Hypothesis 1**

|  | ***Depression (cont. for age)*** | | | ***Depression (cont. for gender)*** | | | ***Depression (cont. for covid risk)*** | | |
| --- | --- | --- | --- | --- | --- | --- | --- | --- | --- |
| *Predictors* | *Estimates* | *CI* | *p* | *Estimates* | *CI* | *p* | *Estimates* | *CI* | *p* |
| (Intercept) | -0.93 | -2.24 – 0.37 | 0.160 | -2.68 | -3.77 – -1.58 | **<0.001** | -2.76 | -4.09 – -1.44 | **<0.001** |
| Time | 0.50 | -0.04 – 1.05 | 0.069 | 0.46 | -0.08 – 1.00 | 0.095 | 0.46 | -0.08 – 1.00 | 0.098 |
| Intolerance of uncertainty | 0.33 | 0.30 – 0.36 | **<0.001** | 0.34 | 0.31 – 0.37 | **<0.001** | 0.34 | 0.31 – 0.37 | **<0.001** |
| Age at T1 | -0.03 | -0.05 – -0.02 | **<0.001** |  |  |  |  |  |  |
| Time x Intolerance of uncertainty | -0.02 | -0.04 – -0.01 | **0.001** | -0.02 | -0.04 – -0.01 | **0.002** | -0.02 | -0.04 – -0.01 | **0.002** |
| Gender (Male) |  |  |  | 0.10 | -0.67 – 0.88 | 0.793 |  |  |  |
| Gender (Other) |  |  |  | 2.52 | -0.46 – 5.50 | 0.097 |  |  |  |
| Gender (Prefer not to say) |  |  |  | -0.95 | -5.51 – 3.61 | 0.683 |  |  |  |
| Covid risk at T1 |  |  |  |  |  |  | 0.05 | -0.28 – 0.39 | 0.762 |
| Marginal R^2^ / Conditional R^2^ | 0.259 / 0.735 | | | 0.251 / 0.735 | | | 0.250 / 0.735 | | |
| ***Table S2.*** Estimates for models predicting depression when controlling for age, gender and covid risk, respectively. *Covid risk was assessed at T1, and is the aggregate score across 5 items, all coded 1 = yes, 0 = no (item 1: Have you ever quarantined? 2: Have you been hospitalised with COVID-19? 3: Do you know anyone diagnosed with COVID-19? 4: Do you know anyone hospitalised with COVID-19? 5: Do you know anyone who has died from COVID-19?).* | | | | | | | | | |

|  | ***Anxiety (cont. for age)*** | | | ***Anxiety (cont. for gender)*** | | | ***Anxiety (cont. for covid risk)*** | | |
| --- | --- | --- | --- | --- | --- | --- | --- | --- | --- |
| *Predictors* | *Estimates* | *CI* | *p* | *Estimates* | *CI* | *p* | *Estimates* | *CI* | *p* |
| (Intercept) | -2.59 | -4.71 – -0.46 | **0.017** | -4.65 | -6.68 – -2.61 | **<0.001** | -5.33 | -7.49 – -3.17 | **<0.001** |
| Time | 0.70 | -0.35 – 1.75 | 0.191 | 0.64 | -0.40 – 1.69 | 0.229 | 0.64 | -0.40 – 1.69 | 0.228 |
| Intolerance of uncertainty | 0.33 | 0.28 – 0.39 | **<0.001** | 0.35 | 0.29 – 0.41 | **<0.001** | 0.35 | 0.29 – 0.41 | **<0.001** |
| Country (UK&USA) | -0.55 | -2.91 – 1.80 | 0.645 | -0.52 | -2.88 – 1.85 | 0.667 | -0.46 | -2.83 – 1.90 | 0.702 |
| Age at T1 | -0.04 | -0.05 – -0.03 | **<0.001** |  |  |  |  |  |  |
| Time x Intolerance of uncertainty | -0.03 | -0.06 – -0.00 | **0.039** | -0.03 | -0.06 – -0.00 | **0.046** | -0.03 | -0.06 – -0.00 | **0.045** |
| Time x Country (UK&USA) | 0.33 | -0.88 – 1.54 | 0.593 | 0.33 | -0.88 – 1.54 | 0.592 | 0.32 | -0.89 – 1.53 | 0.604 |
| Intolerance of uncertainty x Country (UK&USA) | 0.04 | -0.03 – 0.10 | 0.301 | 0.03 | -0.03 – 0.10 | 0.337 | 0.03 | -0.04 – 0.10 | 0.375 |
| Time x Intolerance of uncertainty x Country (UK&USA) | -0.00 | -0.04 – 0.03 | 0.795 | -0.00 | -0.04 – 0.03 | 0.793 | -0.00 | -0.04 – 0.03 | 0.810 |
| Gender (Male) |  |  |  | -0.44 | -1.16 – 0.29 | 0.239 |  |  |  |
| Gender (Other) |  |  |  | 1.34 | -1.44 – 4.12 | 0.344 |  |  |  |
| Gender (Prefer not to say) |  |  |  | 0.56 | -3.69 – 4.80 | 0.796 |  |  |  |
| Covid risk at T1 |  |  |  |  |  |  | 0.26 | -0.05 – 0.58 | 0.098 |
| Marginal R^2^ / Conditional R^2^ | 0.324 / 0.737 | | | 0.312 / 0.738 | | | 0.312 / 0.738 | | |
| ***Table S3.*** Estimates for models predicting anxiety when controlling for age, gender and covid risk, respectively. *Covid risk was assessed at T1, and is the aggregate score across 5 items, all coded 1 = yes, 0 = no (item 1: Have you ever quarantined? 2: Have you been hospitalised with COVID-19? 3: Do you know anyone diagnosed with COVID-19? 4: Do you know anyone hospitalised with COVID-19? 5: Do you know anyone who has died from COVID-19?).* | | | | | | | | | |

**Hypothesis 2**

***Network estimation procedure.*** The combined symptom network of depression and anxiety was estimated with Gaussian Graphical Models (GGM) for each time point and by country (Lauritzen, 1996). Missing data was handled using listwise deletion and only participants with complete data across each item were included. To overcome the potential for false-positive edges resulting from a large number of parameters, we regularised GGMs by applying the Least Absolute Shrinkage and Selection Operator (LASSO; Tibshirani, 1996). This technique employs a penalty approach to control the sum of the partial correlation coefficients by shrinking all edges in the network and reducing small edges exactly to zero (Epskamp and Fried, 2018). We used tuning parameter λ to control the degree of regularisation with extended Bayesian Information Criterion (EBIC) model selection (Foygel & Drton, 2010). The EBIC hyperparameter is typically set between 0 and 0.5 with a higher value generating a more parsimonious estimation (with fewer edges). We set the hyperparameter λ to 0.3 which takes a middle ground between exploration and parsimony when identifying edges in the network.

|  | *Network structure invariance* | | *Global strength invariance* | |
| --- | --- | --- | --- | --- |
|  | *Max edge weight difference* | *p value* | *Difference* | *p value* |
| **Full Sample** | | | | |
| High vs Low IU (T1) | 0.11 | 0.42 | 0.00 | 1.00 |
| High vs Low IU (T2) | 0.17 | 0.61 | **0.52** | **0.02** |
| High vs Low IU (T3) | 0.18 | 0.44 | 0.43 | 0.12 |
| **Full sample High IU** | | | | |
| T1 vs T2 | 0.14 | 0.47 | **0.50** | **0.01** |
| T2 vs T3 | 0.23 | 0.06 | 0.19 | 0.43 |
| T1 vs T3 | 0.14 | 0.49 | 0.32 | 0.08 |
| **Australia High IU** | | | | |
| T1 vs T2 | 0.23 | 0.58 | 0.05 | 0.84 |
| T2 vs T3 | 0.34 | 0.22 | 0.16 | 0.63 |
| T1 vs T3 | 0.27 | 0.37 | 0.21 | 0.38 |
| **USA and UK High IU** | | | | |
| T1 vs T2 | 0.15 | 0.52 | 0.06 | 0.81 |
| T2 vs T3 | 0.22 | 0.20 | 0.03 | 0.92 |
| T1 vs T3 | 0.15 | 0.52 | 0.09 | 0.64 |
| **Full sample Low IU** | | | | |
| T1 vs T2 | 0.12 | 0.90 | 0.01 | 0.93 |
| T2 vs T3 | 0.17 | 0.56 | 0.10 | 0.57 |
| T1 vs T3 | 0.17 | 0.32 | 0.12 | 0.44 |
| **Australia Low IU** | | | | |
| T1 vs T2 | 0.21 | 0.73 | 0.20 | 0.39 |
| T2 vs T3 | 0.19 | 0.99 | 0.13 | 0.74 |
| T1 vs T3 | 0.24 | 0.72 | 0.33 | 0.24 |
| **USA and UK Low IU** | | | | |
| T1 vs T2 | 0.15 | 0.73 | 0.14 | 0.43 |
| T2 vs T3 | 0.20 | 0.53 | 0.13 | 0.59 |
| T1 vs T3 | 0.16 | 0.71 | 0.26 | 0.12 |
| ***Table S4****. Results from the Network Comparison Test, assessing differences in network structure invariance and global strength invariance. Comparisons are made between high and low intolerance of uncertainty across time for the full sample, and for high and low intolerance of uncertainty across time by country. Here the network comprised of depression (PHQ-8) and anxiety (GAD-7) symptoms. Significant differences are highlighted in bold. T1 occurred between May 5, 2020, and September 30, 2020, time 2 between August 5, 2020, and January 29, 2021, and T3 between November 5, 2020, and April 9, 2021. Table S13 includes sample sizes for each sub-group.* | | | | |

**
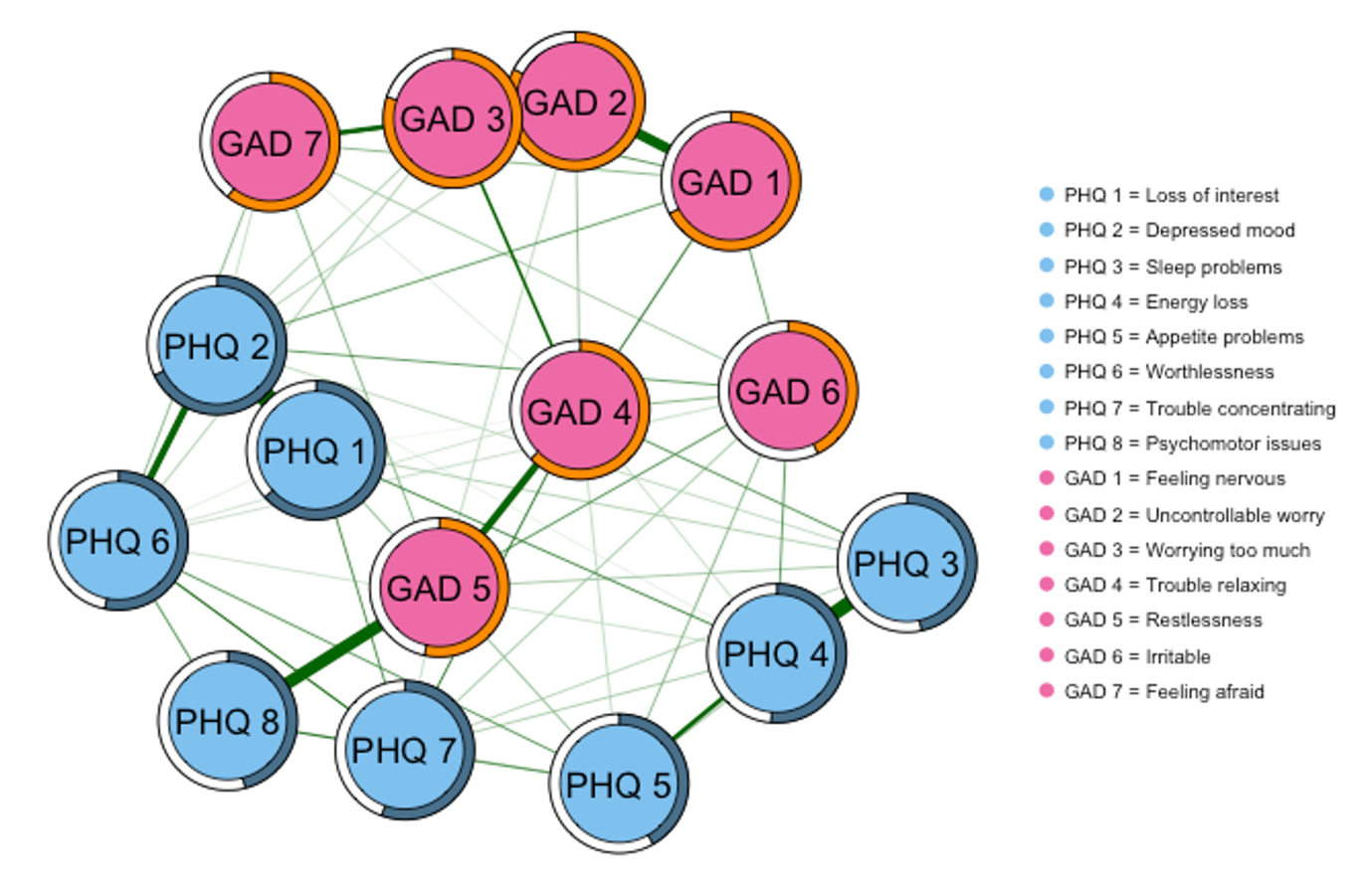
**

***Figure S1.*** *Network diagram of depressive and anxiety symptoms for the full sample at T1. Blue nodes represent depression variables and purple represent anxiety variables.*

|  | ***R^2^*** |
| --- | --- |
| PHQ 1 | 0.63 |
| PHQ 2 | 0.68 |
| PHQ 3 | 0.47 |
| PHQ 4 | 0.51 |
| PHQ 5 | 0.42 |
| PHQ 6 | 0.53 |
| PHQ 7 | 0.56 |
| PHQ 8 | 0.46 |
| GAD 1 | 0.67 |
| GAD 2 | 0.83 |
| GAD 3 | 0.80 |
| GAD 4 | 0.63 |
| GAD 5 | 0.54 |
| GAD 6 | 0.43 |
| GAD 7 | 0.61 |

***Table S5.*** *Node predictability at T1 for the full sample. This is determined by the degree to which each node is predicted by other nodes in the network that share a direct edge. The proportion of variance explained (r^2^) was computed for continuous variables. PHQ-1 = loss of interest, PHQ-2 = depressed mood, PHQ-3= sleep problem, PHQ-4=energy loss, PHQ-5= appetite change, PHQ-6= worthlessness, PHQ-7=trouble concentrating, PHQ-8=psychomotor issues, GAD-1=feeling nervous, GAD-2=uncontrollable worry, GAD-3=worrying too much, GAD-4=trouble relaxing, GAD-5=restlessness, GAD-6=irritable, GAD-7=feeling afraid.*

**Centrality measures.** Centrality measures were computed on the network comprising of depression and anxiety scores at T1 for the full sample. The stability of each centrality measure was computed prior to interpretation. Strength and closeness centrality met the correlation stability-coefficient threshold of 0.5 (Strength = .75; Closeness = .60). However, betweenness centrality was unstable, not meeting this threshold (.44). Therefore, only strength and closeness centrality are reported in figure 4.


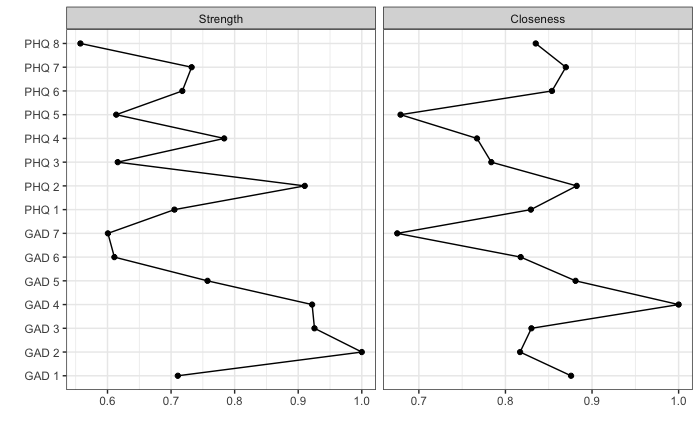


***Figure S2.*** *The relative scores for* *strength and closeness centrality are plotted for each node within the network at T1 for the full sample. Symptom nodes are represented along the Y-axis and the relative centrality scores are represented on the X- axis, from 0-1 (standardised). PHQ-1 = loss of interest, PHQ-2 = depressed mood, PHQ-3= sleep problem, PHQ-4=energy loss, PHQ-5= appetite change, PHQ-6= worthlessness, PHQ-7=trouble concentrating, PHQ-8=psychomotor issues, GAD-1=feeling nervous, GAD-2=uncontrollable worry, GAD-3=worrying too much, GAD-4=trouble relaxing, GAD-5=restlessness, GAD-6=irritable, GAD-7=feeling afraid.*

**Hypothesis 3**

|  | ***Network structure invariance*** | | ***Global strength invariance*** | |
| --- | --- | --- | --- | --- |
|  | *Max edge weight difference* | *p value* | *Difference* | *p value* |
| **Full Sample** |  |  |  |  |
| T1 vs T2 | .09 | .92 | .03 | .90 |
| T2 vs T3 | .14 | .31 | .06 | .78 |
| T1 vs T3 | .14 | .09 | .03 | .91 |
| **Australia** |  |  |  |  |
| T1 vs T2 | .17 | .73 | .26 | .16 |
| T2 vs T3 | .18 | .87 | .13 | .62 |
| T1 vs T3 | .22 | .24 | .12 | .50 |
| **UK & USA** |  |  |  |  |
| T1 vs T2 | .11 | .65 | .06 | .75 |
| T2 vs T3 | .14 | .06 | .13 | .58 |
| T1 vs T3 | **.17** | **.04** | .06 | .76 |
| ***Table S6.*** *Results from the Network Comparison Test, assessing differences in network structure invariance and global strength invariance, across time and by country. Here the network comprised of intolerance of uncertainty (sum score of IUS-12) and depression (PHQ-8) and anxiety (GAD-7) symptoms. Significant differences are highlighted in bold. T1 occurred between May 5, 2020, and September 30, 2020, time 2 between August 5, 2020, and January 29, 2021, and T3 between November 5, 2020, and April 9, 2021.. Table S13 includes sample sizes for each sub-group.* | | | | |

| ***Edge (between symptoms)*** | | ***p value*** | ***Corrected p value*** |
| --- | --- | --- | --- |
| PHQ 1 | PHQ 8 | .003 | .36 |
| PHQ 5 | GAD 1 | .047 | 1 |
| PHQ 1 | GAD 3 | .046 | 1 |
| GAD 3 | GAD 7 | .002 | .24 |
| ***Table S7****. Post-hoc edge weight differences conducted on the edges of a network model comprised of intolerance of uncertainty (sum score of IUS-12) and depression (PHQ-8) and anxiety (GAD-7) symptoms, between T1 and T3 from adults in the UK and USA. Time 1 occurred between May 5, 2020, and September 5, 2020, and time 3 between November 5, 2020, and March 30, 2021. PHQ 1 = Loss of interest, PHQ 5 = Appetite problems, PHQ 8 = Psychomotor problems, GAD 1 = Feeling nervous, GAD 3 = Worrying too much, GAD 7 = Feeling afraid.* | | | |

|  | ***Stability Coefficient*** | | |
| --- | --- | --- | --- |
|  | *Betweenness* | *Closeness* | *Strength* |
| **Full Sample** | | |  |
| T1 | .28 | .67 | **.75** |
| T2 | .13 | .28 | **.67** |
| T3 | .13 | .05 | **.75** |
| **Australia** | | |  |
| T1 | 0 | .13 | .51 |
| T2 | .05 | 0 | .52 |
| T3 | 0 | 0 | .34 |
| **UK & USA** | | |  |
| T1 | .44 | .52 | .75 |
| T2 | 0 | .21 | .44 |
| T3 | .05 | 0 | .67 |
| ***Table S8.*** *Stability Co-efficients for betweenness, closeness and strength centrality, computed on a network model comprised of intolerance of uncertainty (sum score of IUS-12) and depression (PHQ-8) and anxiety (GAD-7) symptoms, across time and by country. Scores above 0.5 are considered good (Epskamp, Borsboom & Fried, 2017). T1 occurred between May 5, 2020, and September 30, 2020, time 2 between August 5, 2020, and January 29, 2021, and T3 between November 5, 2020, and April 9, 2021. Table S13 includes sample sizes for each sub-group.* | | | |

**
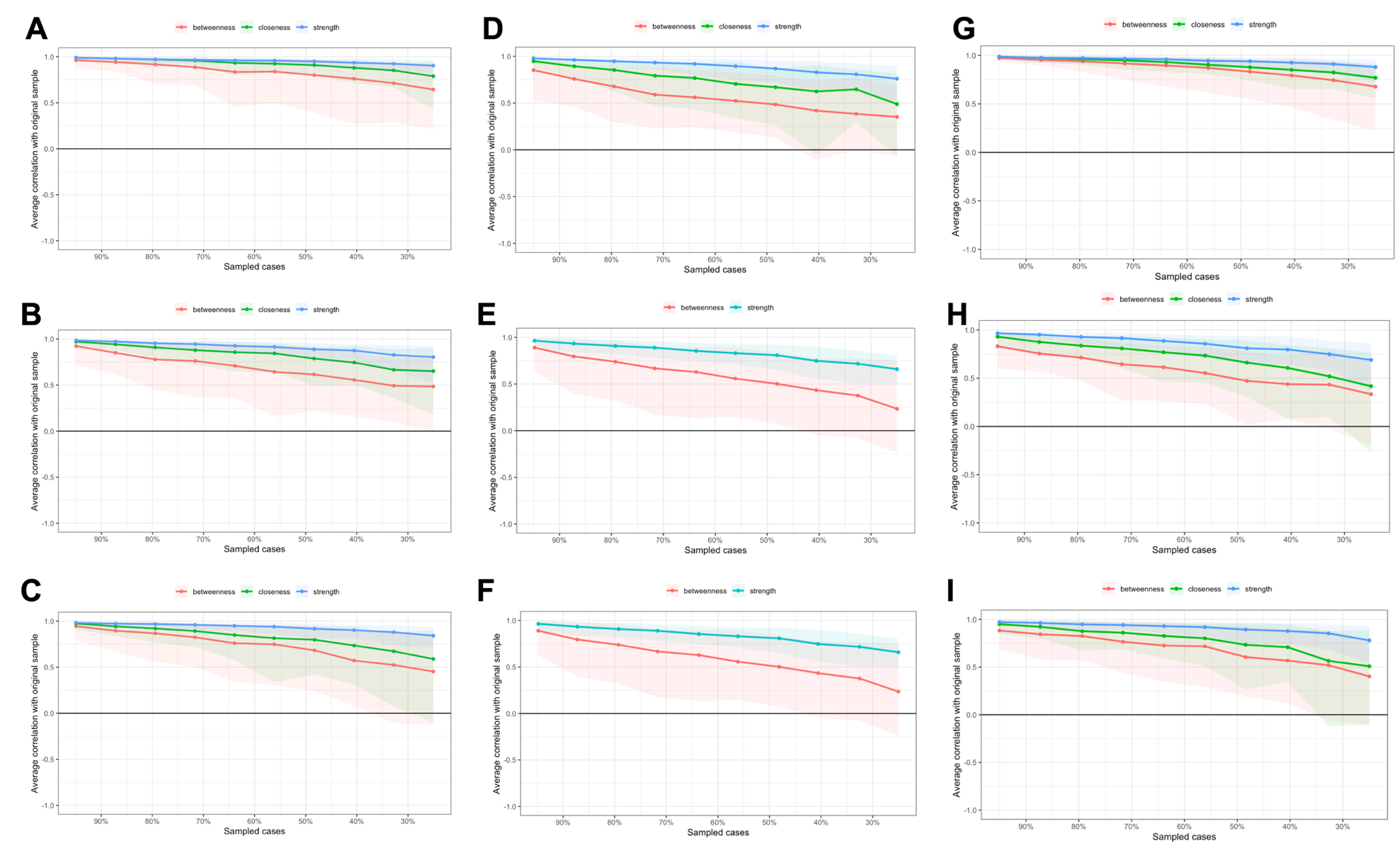
**

**Figure S3:** Stability plots for betweenness, closeness and strength centrality by sample and time point. A-C: Full sample (A= T1; B= T2; C= T3); D-F: Australian sample (D= T1; E= T2; F= T3); G-I: USA/UK sample (G= T1; H= T2; I= T3). T1 occurred between May 5, 2020, and September 30, 2020, time 2 between August 5, 2020, and January 29, 2021, and T3 between November 5, 2020, and April 9, 2021.. Table S13 includes sample sizes for each sub-group.

|  | ***T1 vs T2*** | | ***T2 vs T3*** | | ***T1 vs T3*** | |
| --- | --- | --- | --- | --- | --- | --- |
|  | *Difference* | *p value* | *Difference* | *p value* | *Difference* | *p value* |
| IU | 0.09 | 0.11 | 0.04 | 0.58 | 0.12 | **0.02** |
| PHQ 1 | -0.05 | 0.40 | 0.03 | 0.69 | -0.02 | 0.70 |
| PHQ 2 | 0.05 | 0.34 | -0.14 | **0.03** | -0.09 | 0.12 |
| PHQ 3 | 0.07 | 0.17 | 0.04 | 0.45 | 0.11 | 0.09 |
| PHQ 4 | -0.03 | 0.79 | -0.05 | 0.72 | -0.08 | 0.51 |
| PHQ 5 | -0.05 | 0.34 | -0.02 | 0.78 | -0.07 | 0.22 |
| PHQ 6 | 0.00 | 1.00 | -0.02 | 0.79 | -0.02 | 0.70 |
| PHQ 7 | -0.00 | 0.94 | 0.02 | 0.74 | -0.02 | 0.73 |
| PHQ 8 | -0.06 | 0.68 | 0.05 | 0.68 | -0.01 | 0.98 |
| GAD 1 | 0.01 | 0.83 | -0.08 | 0.25 | -0.07 | 0.21 |
| GAD 2 | -0.05 | 0.34 | 0.07 | 0.31 | 0.02 | 0.70 |
| GAD 3 | 0.09 | 0.15 | -0.11 | 0.08 | -0.03 | 0.62 |
| GAD 4 | 0.10 | 0.15 | -0.08 | 0.29 | 0.02 | 0.81 |
| GAD 5 | -0.81 | 0.22 | -0.03 | 0.79 | -0.12 | 0.19 |
| GAD 6 | 0.02 | 0.68 | 0.04 | 0.57 | 0.06 | 0.42 |
| GAD 7 | -0.5 | 0.39 | 0.14 | 0.06 | 0.09 | 0.13 |
| ***Table S9.*** *Differences in strength centrality of each node, computed on a network model comprised of intolerance of uncertainty (sum score of IUS-12) and depression (PHQ-8) and anxiety (GAD-7) symptoms, from the full sample across time. T1 occurred between May 5, 2020, and September 30, 2020, time 2 between August 5, 2020, and January 29, 2021, and T3 between November 5, 2020, and April 9, 2021. Table S13 includes sample sizes for each sub-group.* | | | | | | |

|  | ***Node predictability (R^2^)*** | | |
| --- | --- | --- | --- |
|  | *T1* | *T2* | *T3* |
| IU | 0.37 | 0.29 | 0.24 |
| PHQ 1 | 0.62 | 0.61 | 0.63 |
| PHQ 2 | 0.71 | 0.70 | 0.73 |
| PHQ 3 | 0.51 | 0.47 | 0.48 |
| PHQ 4 | 0.57 | 0.56 | 0.58 |
| PHQ 5 | 0.47 | 0.48 | 0.50 |
| PHQ 6 | 0.59 | 0.57 | 0.58 |
| PHQ 7 | 0.56 | 0.56 | 0.59 |
| PHQ 8 | 0.41 | 0.46 | 0.45 |
| GAD 1 | 0.73 | 0.68 | 0.72 |
| GAD 2 | 0.83 | 0.83 | 0.81 |
| GAD 3 | 0.82 | 0.79 | 0.91 |
| GAD 4 | 0.70 | 0.66 | 0.73 |
| GAD 5 | 0.56 | 0.56 | 0.56 |
| GAD 6 | 0.52 | 0.49 | 0.51 |
| GAD 7 | 0.65 | 0.63 | 0.56 |
| ***Table S10.*** *Node predictability of each symptom, computed on a network model comprised of intolerance of uncertainty (sum score of IUS-12) and depression (PHQ-8) and anxiety (GAD-7) symptoms, from the full sample across time. Time 1 occurred between May 5, 2020, and September 30, 2020, time 2 between August 5, 2020, and January 29, 2021, and time 3 between November 5, 2020, and April 9, 2021. Table S14 includes sample sizes for each sub-group.* | | | |

| ***Node 1*** | ***Node 2*** | ***Measure*** | ***Lower CI*** | ***Upper CI*** | ***Significant*** |
| --- | --- | --- | --- | --- | --- |
| IU--GAD 7 | IU--PHQ 5 | edge | -0.154654221374777 | -0.0287766015147284 | TRUE |
| IU--GAD 7 | IU--GAD 3 | edge | -0.150096262494612 | 0.0438432993215723 | FALSE |
| IU--GAD 7 | IU--PHQ 2 | edge | -0.16363535640199 | -0.0684452393642583 | TRUE |
| IU--GAD 7 | IU--PHQ 4 | edge | -0.141512174639193 | -0.0118465270358121 | TRUE |
| IU--GAD 7 | IU--PHQ 1 | edge | -0.163602883170073 | -0.0644157254753581 | TRUE |
| IU--GAD 7 | IU--GAD 6 | edge | -0.126968286706186 | 0.0103789481388471 | FALSE |
| IU--GAD 7 | IU--GAD 1 | edge | -0.16085801105313 | -0.0287414115269914 | TRUE |
| IU--GAD 7 | IU--PHQ 8 | edge | -0.161696251333645 | -0.0424314787726945 | TRUE |
| IU--GAD 7 | IU--GAD 4 | edge | -0.16363535640199 | -0.0684452393642583 | TRUE |
| IU--GAD 7 | IU--GAD 5 | edge | -0.163444498899484 | -0.0617752623317125 | TRUE |
| IU--GAD 7 | IU--PHQ 3 | edge | -0.16363535640199 | -0.0674954416779417 | TRUE |
| IU--GAD 7 | IU--PHQ 7 | edge | -0.15745471059084 | -0.0255462068494787 | TRUE |
| IU--GAD 7 | IU--GAD 2 | edge | -0.16363535640199 | -0.0185842877286468 | TRUE |
| IU--GAD 7 | IU--PHQ 6 | edge | -0.141039440186368 | -0.00334327198028616 | TRUE |
| IU--PHQ 5 | IU--GAD 3 | edge | -0.0454972096378982 | 0.13164079140915 | FALSE |
| IU--PHQ 5 | IU--PHQ 2 | edge | -0.0660892849596185 | 0 | FALSE |
| IU--PHQ 5 | IU--PHQ 4 | edge | -0.0486784350131463 | 0.0743403880502724 | FALSE |
| IU--PHQ 5 | IU--PHQ 1 | edge | -0.0660751528082075 | 0.0198410635299678 | FALSE |
| IU--PHQ 5 | IU--GAD 6 | edge | -0.0314607069873423 | 0.0951174842394826 | FALSE |
| IU--PHQ 5 | IU--GAD 1 | edge | -0.0622372283962594 | 0.0571813814540517 | FALSE |
| IU--PHQ 5 | IU--PHQ 8 | edge | -0.0617223804329001 | 0.0450017370102203 | FALSE |
| IU--PHQ 5 | IU--GAD 4 | edge | -0.0660892849596185 | 0 | FALSE |
| IU--PHQ 5 | IU--GAD 5 | edge | -0.0625607057944154 | 0.023125115374711 | FALSE |
| IU--PHQ 5 | IU--PHQ 3 | edge | -0.0660892849596185 | 0 | FALSE |
| IU--PHQ 5 | IU--PHQ 7 | edge | -0.0574086949674218 | 0.062062193117722 | FALSE |
| IU--PHQ 5 | IU--GAD 2 | edge | -0.0635803207800697 | 0.0737076460043791 | FALSE |
| IU--PHQ 5 | IU--PHQ 6 | edge | -0.0435178147209489 | 0.0848594238657628 | FALSE |
| IU--GAD 3 | IU--PHQ 2 | edge | -0.138122253492291 | 0 | FALSE |
| IU--GAD 3 | IU--PHQ 4 | edge | -0.119835432613211 | 0.0714960483495482 | FALSE |
| IU--GAD 3 | IU--PHQ 1 | edge | -0.13736648448999 | 0 | FALSE |
| IU--GAD 3 | IU--GAD 6 | edge | -0.104376807074164 | 0.0872711478102978 | FALSE |
| IU--GAD 3 | IU--GAD 1 | edge | -0.137264097503778 | 0.052173104955315 | FALSE |
| IU--GAD 3 | IU--PHQ 8 | edge | -0.13281856674705 | 0.0353463770941452 | FALSE |
| IU--GAD 3 | IU--GAD 4 | edge | -0.138122253492291 | 0 | FALSE |
| IU--GAD 3 | IU--GAD 5 | edge | -0.137264097503778 | 0 | FALSE |
| IU--GAD 3 | IU--PHQ 3 | edge | -0.138122253492291 | 0 | FALSE |
| IU--GAD 3 | IU--PHQ 7 | edge | -0.130443174848235 | 0.05088481539669 | FALSE |
| IU--GAD 3 | IU--GAD 2 | edge | -0.138122253492291 | 0.0717127282570297 | FALSE |
| IU--GAD 3 | IU--PHQ 6 | edge | -0.113384092855005 | 0.0718118156779656 | FALSE |
| IU--PHQ 2 | IU--PHQ 4 | edge | 0 | 0.0816095683864601 | FALSE |
| IU--PHQ 2 | IU--PHQ 1 | edge | 0 | 0.0293420980687325 | FALSE |
| IU--PHQ 2 | IU--GAD 6 | edge | 0 | 0.10297800381165 | FALSE |
| IU--PHQ 2 | IU--GAD 1 | edge | 0 | 0.0632354826739375 | FALSE |
| IU--PHQ 2 | IU--PHQ 8 | edge | 0 | 0.0549564644028016 | FALSE |
| IU--PHQ 2 | IU--GAD 4 | edge | 0 | 0 | FALSE |
| IU--PHQ 2 | IU--GAD 5 | edge | 0 | 0.0326971179572077 | FALSE |
| IU--PHQ 2 | IU--PHQ 3 | edge | 0 | 0 | FALSE |
| IU--PHQ 2 | IU--PHQ 7 | edge | 0 | 0.0689659293511849 | FALSE |
| IU--PHQ 2 | IU--GAD 2 | edge | 0 | 0.0829696480669676 | FALSE |
| IU--PHQ 2 | IU--PHQ 6 | edge | 0 | 0.0911703216204411 | FALSE |
| IU--PHQ 4 | IU--PHQ 1 | edge | -0.0816095683864601 | 0 | FALSE |
| IU--PHQ 4 | IU--GAD 6 | edge | -0.048720599776499 | 0.0837125103841877 | FALSE |
| IU--PHQ 4 | IU--GAD 1 | edge | -0.0787585862304142 | 0.0472601992845819 | FALSE |
| IU--PHQ 4 | IU--PHQ 8 | edge | -0.0777011902984086 | 0.0366528282144751 | FALSE |
| IU--PHQ 4 | IU--GAD 4 | edge | -0.0816095683864601 | 0 | FALSE |
| IU--PHQ 4 | IU--GAD 5 | edge | -0.0812717627811715 | 0 | FALSE |
| IU--PHQ 4 | IU--PHQ 3 | edge | -0.0816095683864601 | 0 | FALSE |
| IU--PHQ 4 | IU--PHQ 7 | edge | -0.0736725478886612 | 0.0499442385049891 | FALSE |
| IU--PHQ 4 | IU--GAD 2 | edge | -0.0812717627811715 | 0.0507193413041805 | FALSE |
| IU--PHQ 4 | IU--PHQ 6 | edge | -0.0642920820385038 | 0.0660849900701724 | FALSE |
| IU--PHQ 1 | IU--GAD 6 | edge | 0 | 0.10297800381165 | FALSE |
| IU--PHQ 1 | IU--GAD 1 | edge | -0.0251494504808815 | 0.0617232984467733 | FALSE |
| IU--PHQ 1 | IU--PHQ 8 | edge | -0.0251494504808815 | 0.0549564644028016 | FALSE |
| IU--PHQ 1 | IU--GAD 4 | edge | -0.0293420980687325 | 0 | FALSE |
| IU--PHQ 1 | IU--GAD 5 | edge | -0.0266777954353229 | 0.0320195632175711 | FALSE |
| IU--PHQ 1 | IU--PHQ 3 | edge | -0.0293420980687325 | 0 | FALSE |
| IU--PHQ 1 | IU--PHQ 7 | edge | -0.0142443756494899 | 0.0689659293511849 | FALSE |
| IU--PHQ 1 | IU--GAD 2 | edge | -0.0272943661082634 | 0.0829696480669676 | FALSE |
| IU--PHQ 1 | IU--PHQ 6 | edge | 0 | 0.0906240624543815 | FALSE |
| IU--GAD 6 | IU--GAD 1 | edge | -0.0994912629076298 | 0.0217628075749675 | FALSE |
| IU--GAD 6 | IU--PHQ 8 | edge | -0.102867628316239 | 0.0169775371266533 | FALSE |
| IU--GAD 6 | IU--GAD 4 | edge | -0.10297800381165 | 0 | FALSE |
| IU--GAD 6 | IU--GAD 5 | edge | -0.102274648353599 | 0 | FALSE |
| IU--GAD 6 | IU--PHQ 3 | edge | -0.10297800381165 | 0 | FALSE |
| IU--GAD 6 | IU--PHQ 7 | edge | -0.095085090437181 | 0.0330840218096626 | FALSE |
| IU--GAD 6 | IU--GAD 2 | edge | -0.099496059728673 | 0.0350659039059435 | FALSE |
| IU--GAD 6 | IU--PHQ 6 | edge | -0.0771736843811694 | 0.0477926735663922 | FALSE |
| IU--GAD 1 | IU--PHQ 8 | edge | -0.0609236539879664 | 0.0517536532849723 | FALSE |
| IU--GAD 1 | IU--GAD 4 | edge | -0.0632354826739375 | 0 | FALSE |
| IU--GAD 1 | IU--GAD 5 | edge | -0.061311595392285 | 0.0320139789087852 | FALSE |
| IU--GAD 1 | IU--PHQ 3 | edge | -0.0628273257439517 | 0 | FALSE |
| IU--GAD 1 | IU--PHQ 7 | edge | -0.0549352113043597 | 0.0679704068979074 | FALSE |
| IU--GAD 1 | IU--GAD 2 | edge | -0.0632354826739375 | 0.0817048360218704 | FALSE |
| IU--GAD 1 | IU--PHQ 6 | edge | -0.0391231059002206 | 0.0906240624543815 | FALSE |
| IU--PHQ 8 | IU--GAD 4 | edge | -0.0549564644028016 | 0 | FALSE |
| IU--PHQ 8 | IU--GAD 5 | edge | -0.0547297421685019 | 0.0320139789087852 | FALSE |
| IU--PHQ 8 | IU--PHQ 3 | edge | -0.0549508944265044 | 0 | FALSE |
| IU--PHQ 8 | IU--PHQ 7 | edge | -0.0457576344775086 | 0.0676922545431787 | FALSE |
| IU--PHQ 8 | IU--GAD 2 | edge | -0.0549564644028016 | 0.0804280935798145 | FALSE |
| IU--PHQ 8 | IU--PHQ 6 | edge | -0.0339185384448441 | 0.0870408687918537 | FALSE |
| IU--GAD 4 | IU--GAD 5 | edge | 0 | 0.0326971179572077 | FALSE |
| IU--GAD 4 | IU--PHQ 3 | edge | 0 | 0 | FALSE |
| IU--GAD 4 | IU--PHQ 7 | edge | 0 | 0.0689659293511849 | FALSE |
| IU--GAD 4 | IU--GAD 2 | edge | 0 | 0.0829696480669676 | FALSE |
| IU--GAD 4 | IU--PHQ 6 | edge | 0 | 0.0911703216204411 | FALSE |
| IU--GAD 5 | IU--PHQ 3 | edge | -0.0326971179572077 | 0 | FALSE |
| IU--GAD 5 | IU--PHQ 7 | edge | -0.0188302116823265 | 0.0689659293511849 | FALSE |
| IU--GAD 5 | IU--GAD 2 | edge | -0.0321584873280015 | 0.0826586959826324 | FALSE |
| IU--GAD 5 | IU--PHQ 6 | edge | 0 | 0.0905728408677989 | FALSE |
| IU--PHQ 3 | IU--PHQ 7 | edge | 0 | 0.0689659293511849 | FALSE |
| IU--PHQ 3 | IU--GAD 2 | edge | 0 | 0.0826586959826324 | FALSE |
| IU--PHQ 3 | IU--PHQ 6 | edge | 0 | 0.0911703216204411 | FALSE |
| IU--PHQ 7 | IU--GAD 2 | edge | -0.0686194150885249 | 0.0652119031069338 | FALSE |
| IU--PHQ 7 | IU--PHQ 6 | edge | -0.0485757529417974 | 0.0770872870847282 | FALSE |
| IU--GAD 2 | IU--PHQ 6 | edge | -0.0398908784205117 | 0.0902888594565524 | FALSE |

***Table S11.*** *Edge weight differences between intolerance of uncertainty and each other symptom node in the network, computed on a network model comprised of intolerance of uncertainty (sum score of IUS-12) and depression (PHQ-8) and anxiety (GAD-7) symptoms, from the full sample at T1 (between May 5, 2020, and September 30, 2020). Significance set at p<.05 (TRUE).*

| ***Node 1*** | ***Node 2*** | ***Measure*** | ***Lower CI*** | ***Upper CI*** | ***Significant*** |
| --- | --- | --- | --- | --- | --- |
| IU--GAD 7 | IU--PHQ 5 | edge | -0.213504195469016 | 0 | FALSE |
| IU--GAD 7 | IU--GAD 3 | edge | -0.220000075767481 | 0 | FALSE |
| IU--GAD 7 | IU--PHQ 2 | edge | -0.217298750290504 | 0 | FALSE |
| IU--GAD 7 | IU--PHQ 4 | edge | -0.220000075767481 | 0 | FALSE |
| IU--GAD 7 | IU--PHQ 1 | edge | -0.220000075767481 | 0 | FALSE |
| IU--GAD 7 | IU--GAD 6 | edge | -0.204758725376302 | 0.0421978725012183 | FALSE |
| IU--GAD 7 | IU--GAD 1 | edge | -0.218018147199683 | 0 | FALSE |
| IU--GAD 7 | IU--PHQ 8 | edge | -0.217298750290504 | 0 | FALSE |
| IU--GAD 7 | IU--GAD 4 | edge | -0.219345701340192 | 0 | FALSE |
| IU--GAD 7 | IU--GAD 5 | edge | -0.220000075767481 | 0 | FALSE |
| IU--GAD 7 | IU--PHQ 3 | edge | -0.220000075767481 | 0 | FALSE |
| IU--GAD 7 | IU--PHQ 7 | edge | -0.220269399021091 | 0 | FALSE |
| IU--GAD 7 | IU--GAD 2 | edge | -0.220000075767481 | 0 | FALSE |
| IU--GAD 7 | IU--PHQ 6 | edge | -0.212552766973489 | 0.0169898013615832 | FALSE |
| IU--PHQ 5 | IU--GAD 3 | edge | -0.0846373455813699 | 0 | FALSE |
| IU--PHQ 5 | IU--PHQ 2 | edge | -0.0842328417968067 | 0.0848621830362859 | FALSE |
| IU--PHQ 5 | IU--PHQ 4 | edge | -0.0843654623498989 | 0 | FALSE |
| IU--PHQ 5 | IU--PHQ 1 | edge | -0.0846373455813699 | 0 | FALSE |
| IU--PHQ 5 | IU--GAD 6 | edge | -0.0776436537971987 | 0.109323467513845 | FALSE |
| IU--PHQ 5 | IU--GAD 1 | edge | -0.084280166441292 | 0.0526614072103835 | FALSE |
| IU--PHQ 5 | IU--PHQ 8 | edge | -0.0814552559265768 | 0.0627743811428342 | FALSE |
| IU--PHQ 5 | IU--GAD 4 | edge | -0.0846373455813699 | 0.0694419108332635 | FALSE |
| IU--PHQ 5 | IU--GAD 5 | edge | -0.0846373455813699 | 0 | FALSE |
| IU--PHQ 5 | IU--PHQ 3 | edge | -0.0846373455813699 | 0 | FALSE |
| IU--PHQ 5 | IU--PHQ 7 | edge | -0.0854230802084063 | 0 | FALSE |
| IU--PHQ 5 | IU--GAD 2 | edge | -0.0843654623498989 | 0 | FALSE |
| IU--PHQ 5 | IU--PHQ 6 | edge | -0.0792358021751486 | 0.0831693941670241 | FALSE |
| IU--GAD 3 | IU--PHQ 2 | edge | 0 | 0.0916050568707616 | FALSE |
| IU--GAD 3 | IU--PHQ 4 | edge | 0 | 0 | FALSE |
| IU--GAD 3 | IU--PHQ 1 | edge | 0 | 0 | FALSE |
| IU--GAD 3 | IU--GAD 6 | edge | 0 | 0.11254267223914 | FALSE |
| IU--GAD 3 | IU--GAD 1 | edge | 0 | 0.0682649943308722 | FALSE |
| IU--GAD 3 | IU--PHQ 8 | edge | 0 | 0.0715464977198659 | FALSE |
| IU--GAD 3 | IU--GAD 4 | edge | 0 | 0.0709039037964722 | FALSE |
| IU--GAD 3 | IU--GAD 5 | edge | 0 | 0 | FALSE |
| IU--GAD 3 | IU--PHQ 3 | edge | 0 | 0 | FALSE |
| IU--GAD 3 | IU--PHQ 7 | edge | 0 | 0 | FALSE |
| IU--GAD 3 | IU--GAD 2 | edge | 0 | 0 | FALSE |
| IU--GAD 3 | IU--PHQ 6 | edge | 0 | 0.0903049001268194 | FALSE |
| IU--PHQ 2 | IU--PHQ 4 | edge | -0.0913996914169854 | 0 | FALSE |
| IU--PHQ 2 | IU--PHQ 1 | edge | -0.0916050568707616 | 0 | FALSE |
| IU--PHQ 2 | IU--GAD 6 | edge | -0.077316705419588 | 0.109323467513845 | FALSE |
| IU--PHQ 2 | IU--GAD 1 | edge | -0.0898918988486969 | 0.0682649943308722 | FALSE |
| IU--PHQ 2 | IU--PHQ 8 | edge | -0.0877294248244595 | 0.0708822192986644 | FALSE |
| IU--PHQ 2 | IU--GAD 4 | edge | -0.0913996914169854 | 0.0696540040836385 | FALSE |
| IU--PHQ 2 | IU--GAD 5 | edge | -0.0916050568707616 | 0 | FALSE |
| IU--PHQ 2 | IU--PHQ 3 | edge | -0.0916050568707616 | 0 | FALSE |
| IU--PHQ 2 | IU--PHQ 7 | edge | -0.0922976672284948 | 0 | FALSE |
| IU--PHQ 2 | IU--GAD 2 | edge | -0.0916050568707616 | 0 | FALSE |
| IU--PHQ 2 | IU--PHQ 6 | edge | -0.0898918988486969 | 0.0876693009629649 | FALSE |
| IU--PHQ 4 | IU--PHQ 1 | edge | 0 | 0 | FALSE |
| IU--PHQ 4 | IU--GAD 6 | edge | 0 | 0.111652284567942 | FALSE |
| IU--PHQ 4 | IU--GAD 1 | edge | 0 | 0.0682649943308722 | FALSE |
| IU--PHQ 4 | IU--PHQ 8 | edge | 0 | 0.0709163121390068 | FALSE |
| IU--PHQ 4 | IU--GAD 4 | edge | 0 | 0.0709039037964722 | FALSE |
| IU--PHQ 4 | IU--GAD 5 | edge | 0 | 0 | FALSE |
| IU--PHQ 4 | IU--PHQ 3 | edge | 0 | 0 | FALSE |
| IU--PHQ 4 | IU--PHQ 7 | edge | 0 | 0 | FALSE |
| IU--PHQ 4 | IU--GAD 2 | edge | 0 | 0 | FALSE |
| IU--PHQ 4 | IU--PHQ 6 | edge | 0 | 0.0903049001268194 | FALSE |
| IU--PHQ 1 | IU--GAD 6 | edge | 0 | 0.11254267223914 | FALSE |
| IU--PHQ 1 | IU--GAD 1 | edge | 0 | 0.0682649943308722 | FALSE |
| IU--PHQ 1 | IU--PHQ 8 | edge | 0 | 0.0715464977198659 | FALSE |
| IU--PHQ 1 | IU--GAD 4 | edge | 0 | 0.0709039037964722 | FALSE |
| IU--PHQ 1 | IU--GAD 5 | edge | 0 | 0 | FALSE |
| IU--PHQ 1 | IU--PHQ 3 | edge | 0 | 0 | FALSE |
| IU--PHQ 1 | IU--PHQ 7 | edge | 0 | 0 | FALSE |
| IU--PHQ 1 | IU--GAD 2 | edge | 0 | 0 | FALSE |
| IU--PHQ 1 | IU--PHQ 6 | edge | 0 | 0.0903049001268194 | FALSE |
| IU--GAD 6 | IU--GAD 1 | edge | -0.111652284567942 | 0.0258056584623583 | FALSE |
| IU--GAD 6 | IU--PHQ 8 | edge | -0.11080089940159 | 0.0476642221713573 | FALSE |
| IU--GAD 6 | IU--GAD 4 | edge | -0.109323467513845 | 0.039759527872092 | FALSE |
| IU--GAD 6 | IU--GAD 5 | edge | -0.11254267223914 | 0 | FALSE |
| IU--GAD 6 | IU--PHQ 3 | edge | -0.11254267223914 | 0 | FALSE |
| IU--GAD 6 | IU--PHQ 7 | edge | -0.112585164755319 | 0 | FALSE |
| IU--GAD 6 | IU--GAD 2 | edge | -0.11254267223914 | 0 | FALSE |
| IU--GAD 6 | IU--PHQ 6 | edge | -0.110351211557696 | 0.077470425348915 | FALSE |
| IU--GAD 1 | IU--PHQ 8 | edge | -0.06740862699541 | 0.0709163121390068 | FALSE |
| IU--GAD 1 | IU--GAD 4 | edge | -0.0680509702870557 | 0.0709039037964722 | FALSE |
| IU--GAD 1 | IU--GAD 5 | edge | -0.0682649943308721 | 0 | FALSE |
| IU--GAD 1 | IU--PHQ 3 | edge | -0.0682649943308721 | 0 | FALSE |
| IU--GAD 1 | IU--PHQ 7 | edge | -0.0684298548834266 | 0 | FALSE |
| IU--GAD 1 | IU--GAD 2 | edge | -0.0682649943308721 | 0 | FALSE |
| IU--GAD 1 | IU--PHQ 6 | edge | -0.0590869774871034 | 0.0891826507364205 | FALSE |
| IU--PHQ 8 | IU--GAD 4 | edge | -0.0715464977198659 | 0.0680174372549998 | FALSE |
| IU--PHQ 8 | IU--GAD 5 | edge | -0.0715464977198659 | 0 | FALSE |
| IU--PHQ 8 | IU--PHQ 3 | edge | -0.0715464977198659 | 0 | FALSE |
| IU--PHQ 8 | IU--PHQ 7 | edge | -0.0717422723473562 | 0 | FALSE |
| IU--PHQ 8 | IU--GAD 2 | edge | -0.0715464977198659 | 0 | FALSE |
| IU--PHQ 8 | IU--PHQ 6 | edge | -0.0645983671556165 | 0.0888192277262792 | FALSE |
| IU--GAD 4 | IU--GAD 5 | edge | -0.0709039037964722 | 0 | FALSE |
| IU--GAD 4 | IU--PHQ 3 | edge | -0.0709039037964722 | 0 | FALSE |
| IU--GAD 4 | IU--PHQ 7 | edge | -0.0709842474709809 | 0 | FALSE |
| IU--GAD 4 | IU--GAD 2 | edge | -0.0709039037964722 | 0 | FALSE |
| IU--GAD 4 | IU--PHQ 6 | edge | -0.0653247532057977 | 0.0888192277262792 | FALSE |
| IU--GAD 5 | IU--PHQ 3 | edge | 0 | 0 | FALSE |
| IU--GAD 5 | IU--PHQ 7 | edge | 0 | 0 | FALSE |
| IU--GAD 5 | IU--GAD 2 | edge | 0 | 0 | FALSE |
| IU--GAD 5 | IU--PHQ 6 | edge | 0 | 0.0903049001268194 | FALSE |
| IU--PHQ 3 | IU--PHQ 7 | edge | 0 | 0 | FALSE |
| IU--PHQ 3 | IU--GAD 2 | edge | 0 | 0 | FALSE |
| IU--PHQ 3 | IU--PHQ 6 | edge | 0 | 0.0903049001268194 | FALSE |
| IU--PHQ 7 | IU--GAD 2 | edge | 0 | 0 | FALSE |
| IU--PHQ 7 | IU--PHQ 6 | edge | 0 | 0.090945692701873 | FALSE |
| IU--GAD 2 | IU--PHQ 6 | edge | 0 | 0.0903049001268194 | FALSE |

***Table S12.*** *Edge weight differences between intolerance of uncertainty and each other symptom node in the network, computed on a network model comprised of intolerance of uncertainty (sum score of IUS-12) and depression (PHQ-8) and anxiety (GAD-7) symptoms, from the full sample at T2 (between August 5, 2020, and January 29, 2021). Significance set at p<.05 (TRUE).*

| ***Node 1*** | ***Node 2*** | ***Measure*** | ***Lower CI*** | ***Upper CI*** | ***Significant*** |
| --- | --- | --- | --- | --- | --- |
| IU--GAD 7 | IU--PHQ 5 | edge | -0.182470538449151 | 0 | FALSE |
| IU--GAD 7 | IU--GAD 3 | edge | -0.182762829065309 | 0.0651455016970167 | FALSE |
| IU--GAD 7 | IU--PHQ 2 | edge | -0.182762829065309 | 0 | FALSE |
| IU--GAD 7 | IU--PHQ 4 | edge | -0.182470538449151 | 0 | FALSE |
| IU--GAD 7 | IU--PHQ 1 | edge | -0.183595951636872 | 0 | FALSE |
| IU--GAD 7 | IU--GAD 6 | edge | -0.177820271349659 | 0.0420743825244762 | FALSE |
| IU--GAD 7 | IU--GAD 1 | edge | -0.183595951636872 | 0 | FALSE |
| IU--GAD 7 | IU--PHQ 8 | edge | -0.182762829065309 | 0 | FALSE |
| IU--GAD 7 | IU--GAD 4 | edge | -0.183595951636872 | 0 | FALSE |
| IU--GAD 7 | IU--GAD 5 | edge | -0.182470538449151 | 0 | FALSE |
| IU--GAD 7 | IU--PHQ 3 | edge | -0.184211610314255 | 0 | FALSE |
| IU--GAD 7 | IU--PHQ 7 | edge | -0.181989840010964 | 0 | FALSE |
| IU--GAD 7 | IU--GAD 2 | edge | -0.183595951636872 | 0 | FALSE |
| IU--GAD 7 | IU--PHQ 6 | edge | -0.170828117747239 | 0.111716416011692 | FALSE |
| IU--PHQ 5 | IU--GAD 3 | edge | -0.0638726663205895 | 0.132967766449008 | FALSE |
| IU--PHQ 5 | IU--PHQ 2 | edge | -0.0649854915243376 | 0 | FALSE |
| IU--PHQ 5 | IU--PHQ 4 | edge | -0.0639503254507071 | 0.0445382215026281 | FALSE |
| IU--PHQ 5 | IU--PHQ 1 | edge | -0.0649854915243376 | 0 | FALSE |
| IU--PHQ 5 | IU--GAD 6 | edge | -0.0628255141255546 | 0.0852434150492145 | FALSE |
| IU--PHQ 5 | IU--GAD 1 | edge | -0.0649854915243376 | 0.0510838203963543 | FALSE |
| IU--PHQ 5 | IU--PHQ 8 | edge | -0.0639700642311167 | 0.0504891553174043 | FALSE |
| IU--PHQ 5 | IU--GAD 4 | edge | -0.0649854915243376 | 0 | FALSE |
| IU--PHQ 5 | IU--GAD 5 | edge | -0.0649854915243376 | 0 | FALSE |
| IU--PHQ 5 | IU--PHQ 3 | edge | -0.0661459310758614 | 0 | FALSE |
| IU--PHQ 5 | IU--PHQ 7 | edge | -0.0639700642311167 | 0.0501591663883704 | FALSE |
| IU--PHQ 5 | IU--GAD 2 | edge | -0.0649854915243376 | 0 | FALSE |
| IU--PHQ 5 | IU--PHQ 6 | edge | -0.0515623482038994 | 0.138855258469147 | FALSE |
| IU--GAD 3 | IU--PHQ 2 | edge | -0.135205609981982 | 0 | FALSE |
| IU--GAD 3 | IU--PHQ 4 | edge | -0.135205609981982 | 0.0445289409263993 | FALSE |
| IU--GAD 3 | IU--PHQ 1 | edge | -0.13306385053796 | 0 | FALSE |
| IU--GAD 3 | IU--GAD 6 | edge | -0.12998794109339 | 0.0860821868192993 | FALSE |
| IU--GAD 3 | IU--GAD 1 | edge | -0.135205609981982 | 0.051599755514446 | FALSE |
| IU--GAD 3 | IU--PHQ 8 | edge | -0.13306385053796 | 0.0482976586662988 | FALSE |
| IU--GAD 3 | IU--GAD 4 | edge | -0.135205609981982 | 0 | FALSE |
| IU--GAD 3 | IU--GAD 5 | edge | -0.135205609981982 | 0 | FALSE |
| IU--GAD 3 | IU--PHQ 3 | edge | -0.135205609981982 | 0 | FALSE |
| IU--GAD 3 | IU--PHQ 7 | edge | -0.13306385053796 | 0.0501591663883704 | FALSE |
| IU--GAD 3 | IU--GAD 2 | edge | -0.135205609981982 | 0 | FALSE |
| IU--GAD 3 | IU--PHQ 6 | edge | -0.117985335078887 | 0.14237933144142 | FALSE |
| IU--PHQ 2 | IU--PHQ 4 | edge | 0 | 0.0458665537590927 | FALSE |
| IU--PHQ 2 | IU--PHQ 1 | edge | 0 | 0 | FALSE |
| IU--PHQ 2 | IU--GAD 6 | edge | 0 | 0.0880573892872428 | FALSE |
| IU--PHQ 2 | IU--GAD 1 | edge | 0 | 0.0510838203963543 | FALSE |
| IU--PHQ 2 | IU--PHQ 8 | edge | 0 | 0.0506265822783541 | FALSE |
| IU--PHQ 2 | IU--GAD 4 | edge | 0 | 0 | FALSE |
| IU--PHQ 2 | IU--GAD 5 | edge | 0 | 0 | FALSE |
| IU--PHQ 2 | IU--PHQ 3 | edge | 0 | 0 | FALSE |
| IU--PHQ 2 | IU--PHQ 7 | edge | 0 | 0.0501591663883704 | FALSE |
| IU--PHQ 2 | IU--GAD 2 | edge | 0 | 0 | FALSE |
| IU--PHQ 2 | IU--PHQ 6 | edge | 0 | 0.144164450503152 | FALSE |
| IU--PHQ 4 | IU--PHQ 1 | edge | -0.0466348821578642 | 0 | FALSE |
| IU--PHQ 4 | IU--GAD 6 | edge | -0.0429607149927422 | 0.0873747257954283 | FALSE |
| IU--PHQ 4 | IU--GAD 1 | edge | -0.0466348821578642 | 0.051599755514446 | FALSE |
| IU--PHQ 4 | IU--PHQ 8 | edge | -0.0441624545643048 | 0.0482668085262017 | FALSE |
| IU--PHQ 4 | IU--GAD 4 | edge | -0.0466348821578642 | 0 | FALSE |
| IU--PHQ 4 | IU--GAD 5 | edge | -0.0466348821578642 | 0 | FALSE |
| IU--PHQ 4 | IU--PHQ 3 | edge | -0.0500621572633923 | 0 | FALSE |
| IU--PHQ 4 | IU--PHQ 7 | edge | -0.0466348821578642 | 0.0509348170310139 | FALSE |
| IU--PHQ 4 | IU--GAD 2 | edge | -0.0466348821578642 | 0 | FALSE |
| IU--PHQ 4 | IU--PHQ 6 | edge | 0 | 0.139602719673456 | FALSE |
| IU--PHQ 1 | IU--GAD 6 | edge | 0 | 0.0869948453316669 | FALSE |
| IU--PHQ 1 | IU--GAD 1 | edge | 0 | 0.051599755514446 | FALSE |
| IU--PHQ 1 | IU--PHQ 8 | edge | 0 | 0.0506265822783541 | FALSE |
| IU--PHQ 1 | IU--GAD 4 | edge | 0 | 0 | FALSE |
| IU--PHQ 1 | IU--GAD 5 | edge | 0 | 0 | FALSE |
| IU--PHQ 1 | IU--PHQ 3 | edge | 0 | 0 | FALSE |
| IU--PHQ 1 | IU--PHQ 7 | edge | 0 | 0.0509348170310139 | FALSE |
| IU--PHQ 1 | IU--GAD 2 | edge | 0 | 0 | FALSE |
| IU--PHQ 1 | IU--PHQ 6 | edge | 0 | 0.144164450503152 | FALSE |
| IU--GAD 6 | IU--GAD 1 | edge | -0.0880573892872427 | 0.0477934691903268 | FALSE |
| IU--GAD 6 | IU--PHQ 8 | edge | -0.0873747257954283 | 0.0482668085262017 | FALSE |
| IU--GAD 6 | IU--GAD 4 | edge | -0.0880573892872427 | 0 | FALSE |
| IU--GAD 6 | IU--GAD 5 | edge | -0.0880573892872427 | 0 | FALSE |
| IU--GAD 6 | IU--PHQ 3 | edge | -0.0886513993215173 | 0 | FALSE |
| IU--GAD 6 | IU--PHQ 7 | edge | -0.0869948453316669 | 0.0488912486846655 | FALSE |
| IU--GAD 6 | IU--GAD 2 | edge | -0.0880573892872427 | 0 | FALSE |
| IU--GAD 6 | IU--PHQ 6 | edge | -0.0733231019060397 | 0.139151038348541 | FALSE |
| IU--GAD 1 | IU--PHQ 8 | edge | -0.0510838203963543 | 0.0504891553174043 | FALSE |
| IU--GAD 1 | IU--GAD 4 | edge | -0.051599755514446 | 0 | FALSE |
| IU--GAD 1 | IU--GAD 5 | edge | -0.0510838203963543 | 0 | FALSE |
| IU--GAD 1 | IU--PHQ 3 | edge | -0.0589041298507757 | 0 | FALSE |
| IU--GAD 1 | IU--PHQ 7 | edge | -0.051599755514446 | 0.0509348170310139 | FALSE |
| IU--GAD 1 | IU--GAD 2 | edge | -0.051599755514446 | 0 | FALSE |
| IU--GAD 1 | IU--PHQ 6 | edge | 0 | 0.141293845820984 | FALSE |
| IU--PHQ 8 | IU--GAD 4 | edge | -0.0506265822783541 | 0 | FALSE |
| IU--PHQ 8 | IU--GAD 5 | edge | -0.0504891553174042 | 0 | FALSE |
| IU--PHQ 8 | IU--PHQ 3 | edge | -0.0523218950725923 | 0 | FALSE |
| IU--PHQ 8 | IU--PHQ 7 | edge | -0.0497970318243993 | 0.0496285900732287 | FALSE |
| IU--PHQ 8 | IU--GAD 2 | edge | -0.0506265822783541 | 0 | FALSE |
| IU--PHQ 8 | IU--PHQ 6 | edge | -0.00230957231810523 | 0.14237933144142 | FALSE |
| IU--GAD 4 | IU--GAD 5 | edge | 0 | 0 | FALSE |
| IU--GAD 4 | IU--PHQ 3 | edge | 0 | 0 | FALSE |
| IU--GAD 4 | IU--PHQ 7 | edge | 0 | 0.0509348170310139 | FALSE |
| IU--GAD 4 | IU--GAD 2 | edge | 0 | 0 | FALSE |
| IU--GAD 4 | IU--PHQ 6 | edge | 0 | 0.144164450503152 | FALSE |
| IU--GAD 5 | IU--PHQ 3 | edge | 0 | 0 | FALSE |
| IU--GAD 5 | IU--PHQ 7 | edge | 0 | 0.0509348170310139 | FALSE |
| IU--GAD 5 | IU--GAD 2 | edge | 0 | 0 | FALSE |
| IU--GAD 5 | IU--PHQ 6 | edge | 0 | 0.144164450503152 | FALSE |
| IU--PHQ 3 | IU--PHQ 7 | edge | 0 | 0.0520861021255148 | FALSE |
| IU--PHQ 3 | IU--GAD 2 | edge | 0 | 0 | FALSE |
| IU--PHQ 3 | IU--PHQ 6 | edge | 0 | 0.144565013977167 | FALSE |
| IU--PHQ 7 | IU--GAD 2 | edge | -0.0509348170310139 | 0 | FALSE |
| IU--PHQ 7 | IU--PHQ 6 | edge | -0.041496541190551 | 0.141293845820984 | FALSE |
| IU--GAD 2 | IU--PHQ 6 | edge | 0 | 0.144164450503152 | FALSE |

***Table S13.*** *Edge weight differences between intolerance of uncertainty and each other symptom node in the network, computed on a network model comprised of intolerance of uncertainty (sum score of IUS-12) and depression (PHQ-8) and anxiety (GAD-7) symptoms, from the full sample at T3 (between November 5, 2020, and April 9, 2021). Significance set at p<.05 (TRUE).*

|  | *T1* | *T2* | *T3* |
| --- | --- | --- | --- |
| **Hypothesis two** |  |  |  |
| Full sample, high IU | 1082 | 356 | 335 |
| Full sample, low IU | 1002 | 380 | 336 |
| Australia, high IU | 240 | 88 | 75 |
| USA & UK, high IU | 842 | 268 | 260 |
| **Hypothesis three** |  |  |  |
| Full sample | 2084 | 736 | 671 |
| Australia | 546 | 212 | 167 |
| USA & UK | 1538 | 524 | 504 |
| ***Table S14****. Sample sizes for sub-groups in hypothesis two and three.* | | | |

|  | ***GAD 7 (Feeling afraid)*** | | |
| --- | --- | --- | --- |
| *Predictors* | *Estimates* | *CI* | *p* |
| (Intercept) | -1.33 | -1.69 – -0.96 | **<0.001** |
| Intolerance of Uncertainty | 0.07 | 0.06 – 0.08 | **<0.001** |
| Time | 0.01 | 0.00 – 0.02 | **0.013** |
| Intolerance of Uncertainty x Time | -0.00 | -0.00 – -0.00 | **0.007** |
| Observations | 2086 | | |
| R^2^ / R^2^ adjusted | 0.287 / 0.286 | | |
| ***Table S15.*** *Model estimates from a model predicting felling afraid (GAD 7) with an interaction between intolerance of uncertainty and survey completion date (time). Time is represented as the number of days from the first day the survey opened. Omnibus tests revealed a significant main effect of intolerance of uncertainty (F(1,654.86)=830.93, p=<.001) and no significant effect of time (F(1,.14)=.18, p=.67). There was a significant interaction between intolerance of uncertainty and time (F(1,5.77)=7.36, p=.007).* | | | |

**
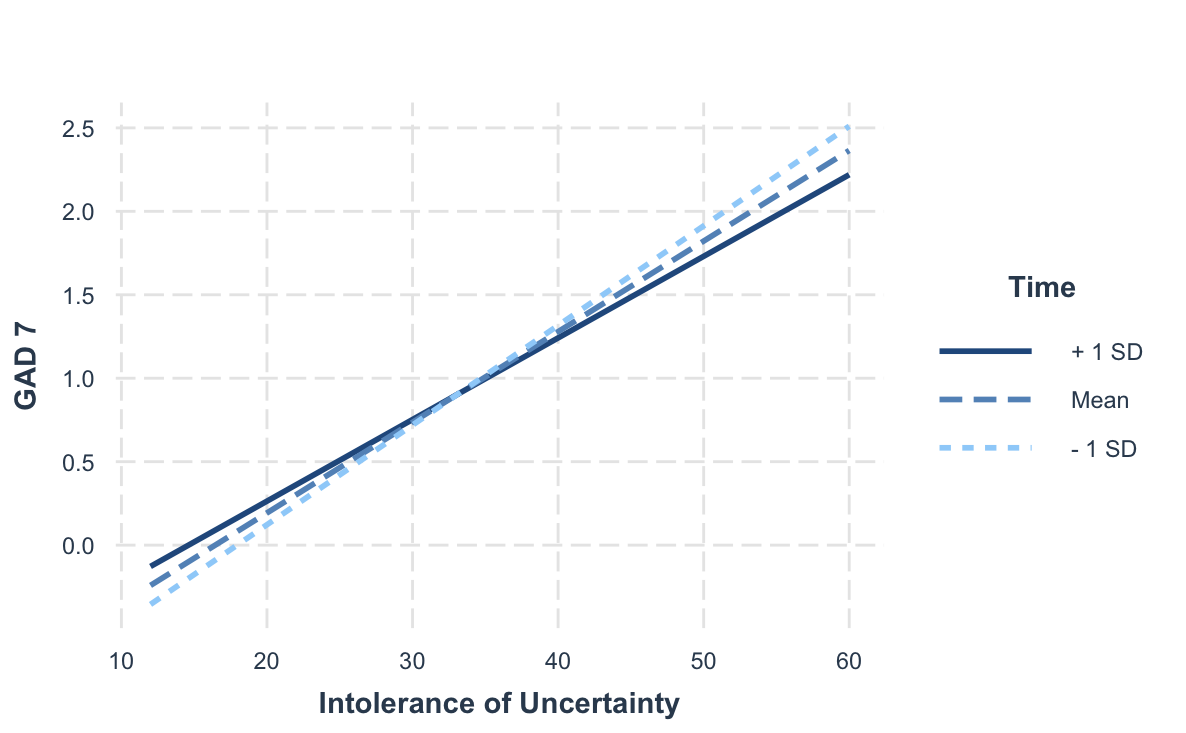
**

***Figure S4.*** *The moderating effect of survey completion date (time) on the relationship between intolerance of uncertainty and feeling afraid (GAD 7). Time is represented as the number of days from the first day the survey opened. Simple slope found a significant relationship between intolerance of uncertainty and GAD 7 at each level of time: -1SD from the mean (Estimate = .06, p<.001), Mean (Estimate = .05, p<.001), +1SD (Estimate = .05, p <.001).*
